# Turbulent particle pair diffusion: Numerical simulations

**DOI:** 10.1371/journal.pone.0216207

**Published:** 2019-05-20

**Authors:** Nadeem A. Malik

**Affiliations:** Department of Mathematics and Statistics, King Fahd University of Petroleum and Minerals, Dhahran, Saudi Arabia; Coastal Carolina University, UNITED STATES

## Abstract

A theory for turbulent particle pair diffusion in the inertial subrange [Malik NA, PLoS ONE 13(10):e0202940 (2018)] is investigated numerically using a Lagrangian diffusion model, Kinematic Simulations [Kraichnan RH, Phys. Fluids 13:22 (1970); Malik NA, PLoS ONE 12(12):e0189917 (2017)]. All predictions of the theory are observed in flow fields with generalised energy spectra of the type, *E*(*k*) ∼ *k*^−*p*^. Most importantly, two non-Richardson regimes are observed: for short inertial subrange of size 10^2^ the simulations yield quasi-local regimes for the pair diffusion coefficient, $K\left(l\right)∼\sigma_{l}^{(1+p)/2}$; and for asymptotically infinite inertial subrange the simulations yield non-local regimes $K\left(l\right)∼\sigma_{l}^{\gamma }$, with *γ* intermediate between the purely local scaling *γ*^*l*^ = (1 + *p*)/2 and the purely non-local scaling *γ*^*nl*^ = 2. For intermittent turbulence spectra, *E*(*k*) ∼ *k*^−1.72^, the simulations yield $K∼\sigma_{l}^{1.556}$, in agreement with the revised 1926 dataset $K∼\sigma_{l}^{1.564}$ [Richardson LF, Proc. Roy. Soc. Lond. A 100:709 (1926); Malik NA, PLoS ONE 13(10):e0202940 (2018)]. These results lend support to the physical picture proposed in the new theory that turbulent diffusion in the inertial subrange is governed by both local and non-local diffusion transport processes.

## 1 Introduction

Turbulent transport and mixing play an essential role in many natural and industrial processes [1–9], where concentration fluctuations, which is related to the pair separation, often play a critical role. Most theories of turbulent particle pair diffusion assume Richardson’s locality hypothesis [10, 11]. However, a new theory for turbulent particle pair diffusion based on the physical picture that both local and non-local diffusional processes govern the pair diffusion process has been proposed by the author in [12].

The main prediction of the new theory in [12] is the existence of two non-Richardson regimes in inertial subranges with generalised energy spectra *E*(*k*) ∼ *k*^−*p*^: a quasi-local (i.e. approximately local) regime at moderate inertial subrange size, *R*_*k*_ ≪ ∞, where $K\left(l,p\right)\approx \sigma_{l}^{(1+p)/2}$; and a non-local regime at asymptotically infinite inertial subrange size, *R*_*k*_ → ∞, where $K\left(l,p\right)∼\sigma_{l}^{\gamma (p)}$ and < (1 + *p*)/2 < *γ*(*p*) < 2, and 1 < *p* ≤ 3.

The power laws, *γ*(*p*), cannot be determined from the theory alone. For this, and for investigating other predictions of the theory, ideally we would need experiments or Direct Numerical Simulations which resolves all the time and length scales in the turbulence. At the current time the capabilities of both experiments and DNS are far from being able to examine large inertial subranges. Nevertheless, we can make some progress through the use of diffusion models.

Here, the aim is to investigate the predictions of the new theory numerically using Kinematic Simulations (KS) which is a Lagrangian diffusion model and it has the advantage that it can generate very large inertial subranges which is essential to test the new theory. KS has been used extensively in the past for pair diffusion studies (see Section 3 below). KS can be indicative of the statistical scaling laws in the inertial subrange.

Dissipation range pair diffusion, where the initial particle separation is such that *l*_0_ ≪ *η*, is outside the scope of the present work. A theory for dissipation range release would likely yield different scaling laws because the ensemble of particle pairs would attain a distribution of separations and enter the inertial subrange at different times after release. The subsequent inertial range diffusion would in effect be an average over different virtual release times. Furthermore, in the present KS we do not pose a dissipation range energy spectrum which is essential for investigating dissipation scale diffusion.

In Section 2 we summaries the new theory developed in [12], and in Section 3 we describe the KS method. In Section 4.1 we describe the simulation results for large inertial subranges of size *R*_*k*_ = 10^6^. In Section 4.2 the results for small inertial subranges *R*_*k*_ = 10^2^ are described. In Section 4.3 the simulation results for the transition in the scaling laws in *K* as the size of the inertial subrange increases from small to very large values are described. In Section 5, cases for very small initial separations *l*_0_ ≪ *η* in order to isolate and expose the non-local diffusional process is investigated. In Section 6 we estimate the numerical errors in the results. We discuss and draw conclusions from the results, and speculate its significane to the general theory of turbulence in Section 7.

## 2 Summary of the new non-local theory

In order to characterize the pair diffusion process, Richardson assumed a scale dependent pair diffusion coefficient (turbulent diffusivity), because convective gusts of winds increase the pair separation at different rates depending on the separation [13–15]. In 1926, from observational data of turbulent pair diffusion coefficients collected from different sources, he assumed an approximate constant power law fit to the data, *K*(*l*) ∼ *l*^4/3^, [10]. This is equivalent to 〈*l*^2^〉 ∼ *t*^3^ [11, 16], and is often referred to as the Richardson-Obukov *t*^3^-regime. *l*(*t*) is the pair separation at time *t* and the angled brackets is the ensemble average over particle pairs. Note that the assumed 4/3-scaling is consistent with Kolmogorov turbulence K41 theory, see [12].

However, to date the validity of Richardson’s scaling law has not been established. The general consensus among scientists in the field at the current time is that the collection of observational data, experimental data, and Direct Numerical Simulation, suggests a convergence towards a Richard-Obukov locality scaling, but as noted by Salazar and Collins, “.. there has not been an experiment that has unequivocally confirmed R-O scaling over a broad-enough range of time and with sufficient accuracy” [17]

Furthermore, in [12] it was noted that one of the data-points used in Richardson comes from molecular diffusion studies and should be dis-regarded. The remaining data-points are sound, coming from geophysical turbulence settings containing extended inertial subranges. The line of best fit to this improved dataset displays an unequivocal non-local scaling, *K* ∼ *l*^1.564^; see Fig 1 in [12].

This indicates that non-local diffusional processes cannot be ignored *a priori* in a general theory of turbulent pair diffusion, which motivates the development of the present local-non-local theory.

### 2.1 The new theory

The problem is to determine the pair diffusion coefficient (diffusivity), *K* = 〈**l** · **v**〉, of an ensemble of pairs of fluid particles in a field of homogeneous turbulence with an energy density spectrum, *E*(*k*) containing an generalised inertial subrange, *E*(*k*) ∼ *k*^−*p*^, *k*_1_ ≤ *k* ≤ *k*_*η*_, for 1 < *p* ≤ 3, and such that *E*(*k*) → 0 as *k* → 0. The particles in a pair are located at **x**_1_(*t*) and **x**_2_(*t*) at time *t*, the pair displacement vector is **l**(*t*) = **x**_2_(*t*) − **x**_1_(*t*), and the pair separation is $l\left(t\right)=\sqrt{l_{1}^{2}+l_{2}^{2}+l_{3}^{2}}=\left|x_{2}\left(t\right)-x_{1}\left(t\right)\right|$. The initial separation at some earlier time, *t*_0_, is denoted by *l*_0_ = |**x**_2_(*t*_0_) − **x**_1_(*t*_0_)|. The turbulent velocity field is, **u**(**x**, *t*), and the particle velocities at time t are, respectively, **u**_1_(*t*) = **u**(**x**_1_(*t*), *t*) and **u**_2_(*t*) = **u**(**x**_2_(*t*), *t*), and the pair relative velocity is **v**(*t*) = **u**_2_(*t*) − **u**_1_(*t*).

We assume point source release, which in practical terms means that the initial pair separation must be close to the Kolmogorov length scale, *l*_0_ ≈ *η*. Without loss of generality, it will also be assumed that, *t*_0_ = 0.

We define the size of the inertial subrange *R*_*k*_ to be,

$$\begin{array}{ccc} R_{k} & = & \frac{k_{\eta }}{k_{1}} \end{array}$$ (1)

where the inertial subrange part of the energy spectrum *E*(*k*) is defined in the wavenumber range *k*_1_ ≤ *k* ≤ *k*_*η*_.

We follow the usual convention and evaluate *K* at typical values of, *l*, namely at $l∼\sigma_{l}=\sqrt{\langle l^{2}\rangle }$. Thus, the locality scaling is replaced by, $K\left(l\right)∼\sigma_{l}^{4/3}$.

The theory is developed through a novel mathematical approach of decomposing the pair relative velocity, **v**(**l**) = *d***l**/*dt*, as a Fourier integral. Assuming homogeneity, the ensemble average of the scalar product of **l** with **v** yields a Fourier decomposition of the diffusion coefficient itself,

$$\begin{array}{ccc} K(l)∼\langle l\cdot v\rangle & ∼ & \int \left\langle \left(l\cdot A\right)\left[\exp(ik\cdot l)-1\right]\right\rangle d^{3}k. \end{array}$$ (2)

where the integration is over the range of turbulent wavenumbers, and **A**(**k**) is the Fourier coefficient of the Eulerian velocity field **u**(**x**).

Let *k*_*l*_ = 1/*l* be the pair separation wavenumber, so that

$$\begin{array}{ccc} k_{l}\left(t\right) & ∼ & \frac{1}{\sigma_{l}\left(t\right)}. \end{array}$$ (3)

Note that *k*_*l*_(*t*) changes with time.

The new theory assumes the existence of two broadly independent diffusional processes within the inertial subrange that contribute to the pair diffusion process as a whole. The two transport processess are correlated locally and non-locally in space, respectively. This can also be expressed in terms of equivalent wavenumbers; thus, each transport process acts from its own range of wavenumbers relative to *k*_*l*_, labeled **l** (local) and **nl** (non-local):

**l**: a local diffusion process operates at wavenumbers that are local to *k*_*l*_, say in the range $\left[k_{l}^{*},k_{l}\right]$ and such that *k* ≈ *k*_*l*_, and |**k** · **l**| ≈ 1. Within this range of wavenumbers local scalings apply.
**nl**: a non-local diffusion process operates at wavenumbers that are non-local to *k*_*l*_, say in the range in the range $\left[k_{1},k_{l}^{*}\right]$, and such that *k* ≪ *k*_*l*_, and |**k** · **l**| ⪡ 1. Non-local scalings apply in this range of wavenumbers.

$k_{l}^{*}$ is some arbitrary wavenumber that separates the two processes, and $k_{1}<k_{l}^{*}<k_{l}$. (A third transport process at very small wavenumbers *k* ≪ *k*_*l*_ has been shown to be negligible, [12]).

This implies that the integral in Eq (2) is the sum of two integrals over different wavenumber ranges which are defined as follows:

$$\begin{array}{ccc} K(l) & ∼ & (\int_{nl}+\int_{l})\left\langle \left(l\cdot A\right)\left(\exp(ik\cdot l)-1\right)\right\rangle \;d^{3}k \end{array}$$ (4)

which we rephrase as,

$$\begin{array}{ccc} K(l) & ∼ & K^{nl}+K^{l}. \end{array}$$ (5)

We assume a generalised inverse power-law energy spectrum in the inertial subrange in the swept frame of reference,

$$\begin{array}{ccc} E(k) & = & C_{k}\varepsilon^{2/3}L^{5/3-p}k^{-p},\;k_{1}<k<k_{\eta },\;1<p\leq 3 \end{array}$$ (6)

where *C*_*k*_ is a constant. A length scale *L* is necessary for dimensional consistency. *L* scales with some length scale that is characteristic of the large energy scales, such as the integral length scale, or the Taylor length scale. With this spectrum, and some closure assumptions detailed in [12], Eq (4) becomes,

$$\begin{array}{ccc} K(l,p) & ∼ & \varepsilon^{1/3}L^{(5/3-p)/2}(\int_{nl}k^{(1-p)/2}dk+F_{l}\int_{l}k^{(1-p)/2}dk)\sigma_{l}^{2} \end{array}$$ (7)

The locality scaling is obtained by removing the non-local integral in this equation. This yields,

$$\begin{array}{ccc} K^{l}\left(l,p\right) & ∼ & F_{l}\varepsilon^{1/3}L^{(5/3-p)/2}\sigma_{l}^{\gamma^{l}},\;\text{where}\;\gamma^{l}\left(p\right)=\left(1+p\right)/2,\;1<p\leq 3 \end{array}$$ (8)

and *F*_*l*_ < 1 is a constant. For Kolmogorov turbulence, *p* = 5/3, this gives, $K∼\sigma_{l}^{4/3}$, which recovers the Richardson’s 4/3-scaling law, [18–20], which validates the derivation.

The non-local scaling in the first integral can be obtained in a similar manner. If the upper end of the inertial subrange is assumed to scale with the large scales, *k*_1_ ∼ 1/*L*, then this yields,

$$\begin{array}{ccc} K^{nl}\left(l,p\right) & ∼ & S_{nl}\varepsilon^{1/3}L^{-2/3}\sigma_{l}^{\gamma^{nl}},\;\text{with}\;\gamma^{nl}\left(p\right)=2,\;1<p\leq 3 \end{array}$$ (9)

*γ*^*nl*^(*p*) is the non-locality scaling and it is always equal to 2 independent of *p*. This corresponds to strain dominated motion with $K^{nl}∼\sigma_{l}^{2}$. *S*_*nl*_ is a constant.

The overall expression for the turbulent pair diffusion coefficient is therefore,

$$\begin{array}{ccc} K(l,p) & ∼ & O(F_{l}\varepsilon^{1/3}L^{(5/3-p)/2}\sigma_{l}^{\gamma^{l}})+O(S_{nl}\varepsilon^{1/3}L^{-2/3}\sigma_{l}^{2}),\;1<p\leq 3, \end{array}$$ (10)

or simply,

$$\begin{array}{ccc} K(l,p) & ∼ & O(\sigma_{l}^{\gamma^{l}})+O(\sigma_{l}^{\gamma^{nl}}),\;1<p\leq 3 \end{array}$$ (11)

$$\begin{array}{ccc} \text{where}\;\gamma^{l}\left(p\right) & = & (1+p)/2\;\;\text{local}\;\text{scaling} \end{array}$$ (12)

$$\begin{array}{ccc} \gamma^{nl}\left(p\right) & = & 2\;\;\;\;\text{non-local}\;\text{scaling} \end{array}$$ (13)

To complete the analysis we need to obtain an experssion for the balance of the local and non-local diffusion terms, defined to be ratio,

$$\begin{array}{ccc} M_{K}\left(p,R_{l},C\right) & = & \frac{K^{nl}}{K^{l}}∼\frac{1}{F_{l}}(\frac{1}{C^{\left(3-p)/2\right)}}-\frac{1}{R_{l}^{\left(3-p)/2\right)}}) \end{array}$$ (14)

The quantity

$$\begin{array}{ccc} R_{l}\left(t\right) & = & \frac{k_{l}\left(t\right)}{k_{1}} \end{array}$$ (15)

is the size of the inertial subrange relative to the pair separation in wavenumber space. The quantity *C* is,

$$\begin{array}{ccc} C(p,R_{l}) & = & \frac{k_{l}}{k_{l}^{*}}. \end{array}$$ (16)

*C* is an important quantity, which we call the locality kernel because it defines the extent (or size), relative to the pair separation, where local scaling is expected to dominate.

In [12] it was shown that under fairly weak restrictions on *C*—essentially little more than smoothness of *C* as a fucntion of *p* and *R*_*l*_—over a wide range of smooth test functions for *C*, in the critical range of *p* close to Kolmogorov’s *p* = 5/3 the balance of local and non-local diffusional processes is close to unity,

$$\begin{array}{ccc} M_{K} & \approx & O(1)\;\text{close}\;\text{to}\;p=5/3. \end{array}$$ (17)

This indicates that the assumption that both local and non-local diffusional processses are significant is physically reasonable. (*F*_*l*_ was chosen to be *F*_*l*_ = 0.25 in Eq (14)).

We assume an extension of Richardson’s second hypothesis, that in the limit of infinite inertial subrange, for every *p* the diffusion coefficient is described by a single power, say *γ*(*p*), across all scales. Thus, from Eq (11) *K*(*l*, *p*) must be a power law scaling which is intermediate between the local and non-local scalings,

$$\begin{array}{ccc} K(l,p) & ∼ & \sigma_{l}^{\gamma (p)},\;\text{as}\;R_{k}/C\to \infty \;\text{with}\;\gamma^{l}\left(p\right)<\gamma \left(p\right)<\gamma^{nl}\left(p\right),\;1<p<3. \end{array}$$ (18)

Only in some special asymptotic limits do we obtain significantly different behaviour. Firstly, as *p* → 1 then *M*_*K*_ ≪ 1, and therefore *K*^*nl*^ ≪ *K*^*l*^, yielding the locality limit,

$$\begin{array}{ccc} K(l,p) & \to & K^{l}\left(l,1\right)∼\sigma_{l}^{1}\;\text{as}\;p\to 1. \end{array}$$ (19)

Secondly, as *p* → 3 then *M*_*K*_ ≫ 1, and therefore *K*^*nl*^ ≫ *K*^*l*^, yielding the non-locality limit,

$$\begin{array}{ccc} K(l,p) & \to & K^{nl}\left(l,3\right)∼\sigma_{l}^{2}\;\text{as}\;p\to 3. \end{array}$$ (20)

Thus, as *p* → 1 then *γ*(*p*) → 1; and as *p* → 3 then *γ*(*p*) → 2. Furthermore, *γ*(*p*) must transform smoothly between these limiting cases as *p* goes from 1 to 3. Globally, 1 < *γ*(*p*) ≤ 2.

Eq (19) is the main prediction of the new theory, and it is equivalent to the mean square separation scaling,

$$\begin{array}{ccc} \left\langle l^{2}\right\rangle & ∼ & t^{\chi (p)},\;\text{where}\;\chi \left(p\right)=\frac{2}{2-\gamma (p)} \end{array}$$ (21)

This is a non-linear relation between *γ* and *χ*, so small changes in *γ* could produce large changes in *χ*.

Some specific results can be obtained from these sclaing laws. For Kolmogorov turbulence, *E* ∼ *k*^−5/3^, the new theory predicts that, *γ* > 4/3, and *χ* > 3.

For turbulence with intermittency *μ*_*I*_ > 0, such that *E* ∼ *k*^−(5/3+*μ*_*I*_)^, the scaling is,

$$\begin{array}{ccc} \gamma_{\mu_{I}} & >\gamma_{\mu_{I}}^{l} & =4/3+\mu_{I}/2. \end{array}$$ (22)

**Fig 1:**
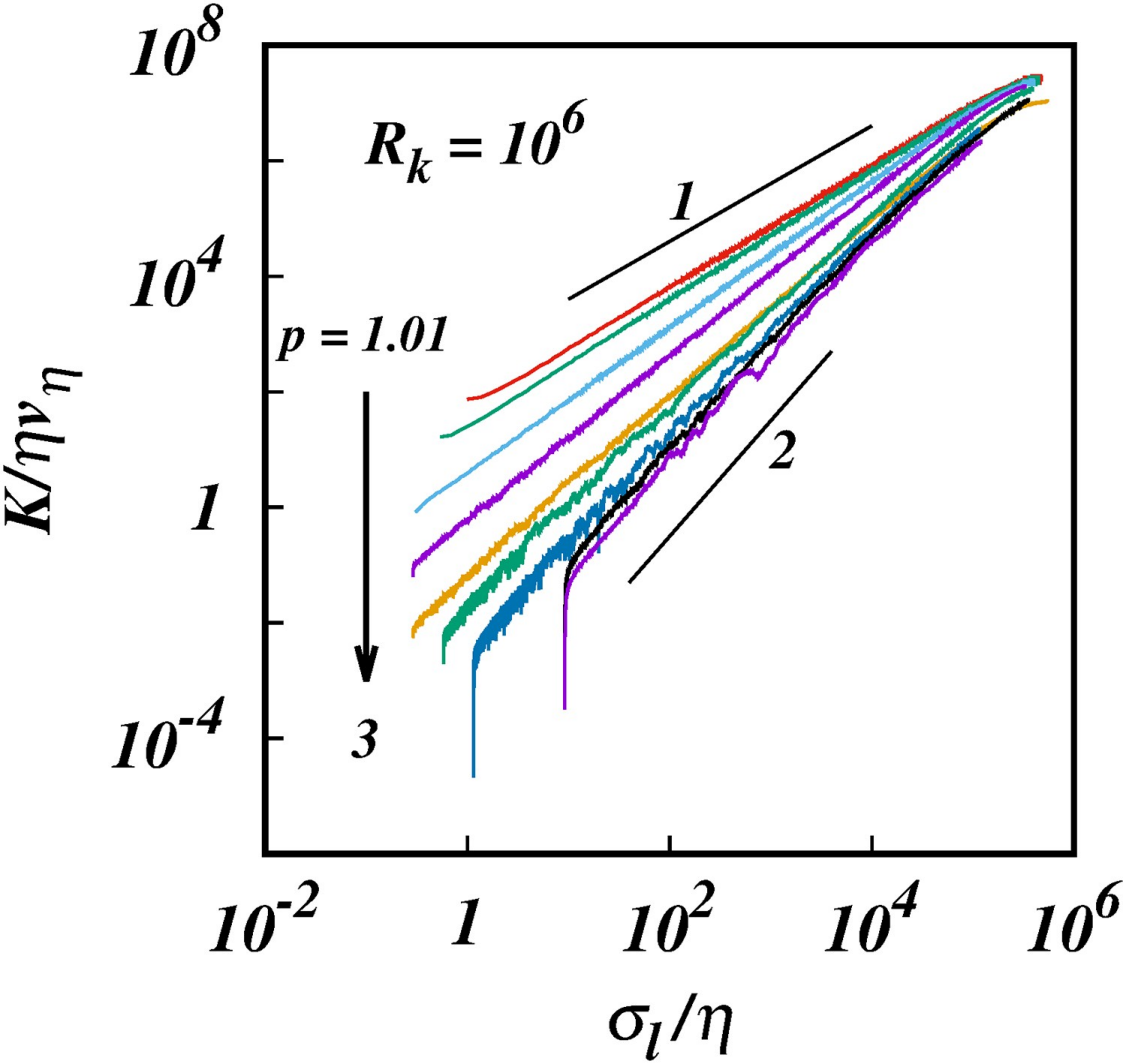
The turbulent pair diffusion coefficient, log(*K*/*ηv*_*η*_), against, log(*σ*_*l*_/*η*), from KS with energy spectra, *E*(*k*) ∼ *k*^−*p*^, and inertial subrange size, *R*_*k*_ = 10^6^. For clarity only 10 of the 25 cases in Table 1 are shown: *p* = 1.01, 1.1,1.3, 1.5, 5/3, 1.9,2.2, 2.5, 2.9, 3.0. Solid black lines of slopes 1 and 2 are shown for comparison.

Under the locality assumption, in real turbulence with intermittency *p* = 1.72 we should obtain the scaling *γ*^*l*^ = 1.36, and *χ*^*l*^ = 3.125. Thus, the classical RO-*t*^3^ regime does not actually exist! Richardson’s 1926 dataset, Fig 1, from real geophysical turbulence (i.e. including intermittency) suggests a scaling of, $\gamma_{\mu_{I}}\approx 1.564$, i.e. $K_{\mu_{I}}∼\sigma_{l}^{1.564}$.

A non-local 4/3-law, $K∼\sigma_{l}^{4/3}$, is precited for some spectrum, $E∼k^{-p_{*}}$, where p_*_ < 5/3 and *γ*(p_*_) = 4/3. This is equivalent to 〈*l*^2^〉 ∼ *t*^3^, with *χ*(p_*_) = 3, which is a new non-Richardson-Obukhov *t*^3^-regime for the mean square separation.

We define *M*_*γ*_(*p*) to be the ratio of the scaling power *γ*(*p*) to and local scaling powers *γ*^*l*^(*p*),

$$\begin{array}{ccc} M_{\gamma }\left(p\right) & = & \frac{\gamma (p)}{\gamma^{l}\left(p\right)}. \end{array}$$ (23)

*M*_*γ*_(*p*) is equal to 1 at both *p* = 1 and *p* = 3, and since *M*_*γ*_ > 1 in the range 1 < *p* < 3, then there must be a maximum in *M*_*γ*_ at some *p* = *p*_*m*_ for an energy spectrum $E∼k^{-p_{m}}$, where 1 < *p*_*m*_ < 3.

The sketches in Figs 13 and 14 in [12] summarise the preditions from the new theory for, respectively, the non-local regimes in the limit of asymptotically infinite inertial subranges *R*_*k*_ → ∞, and the quasi-local regimes in the limit of short finite inertial subranges *R*_*k*_ ≈ 10^2^.

Finally, we remark that, ultra-violet corrections from the high wavenumber close to *k*_*η*_, and infra-red corrections from the low wavenumbers close to *k*_1_ may modifying some of the scalings law specially in the limit of small inertial subranges.

### 2.2 Exposing the local and non-local processes

It may be possible to isolate the individual local and non-local processes in different limts. From the expression in Eq 14 it is evident that the analysis is valid only if *R*_*l*_ > *C*. The non-local process, which is the first term in Eq (11), exists in the wavenumber range $\left[k_{1},k_{l}^{*}\right]$; but suppose that the inertial subrange itself is so small that it is close to the size of the locality kernel itself, *R*_*k*_ ≈ *C*? This corresponds to taking the limit *R*_*l*_/*C* → 1 in Eq (14), which implies that *M*_*k*_ → 0 which is a locality dominated limit. Thus quasi-local diffusion regimes can be recovered in the non-Richardson limit of small but finite inertial subrange because of the absence of non-local processes,

$$\begin{array}{ccc} K(l,p) & \approx & \sigma_{l}^{(1+p)/2},\;\text{for}\;R_{k}\approx C \end{array}$$ (24)

As we progressively increase the size of the inertial subrange we would expect to see a smooth transition from the locality regime at moderate inertial subrange to the non-locality regime at very large (effectively infinite) subrange, as illustrated in the sketch in Fig 15 in [12].

We can also ‘turn off’ the local process in Eq (11) by simply removing the ‘local’ part of the specturm—this is equivalent to taking a very small initial separation *l*_0_ ≪ *η*. Then there is a spectral gap between the *k*_0_ = 1/*l*_0_ and *k*_*η*_ ≪ *k*_0_ where *E*(*k*) = 0. If this gap is large enough then the spectrum between *k*_1_ ≤ *k* ≤ *k*_*η*_ will in efffect be non-local to the pair separation process so long as *σ*_*l*_(*t*) ≪ *η*, and we should then observe pure strain dominated pair separation

$$\begin{array}{ccc} K(l,p) & ∼ & \sigma_{l}^{2},\;\text{for}\;\sigma_{l}\ll \eta \end{array}$$ (25)

for all *p*, which is equivalent to exponential growth in time, *σ*_*l*_(*t*) ∼ *l*_0_ exp(*St*), where *S* depends upon the form of *E*(*k*).

In the next sections we examine the predictions of the new theory using Kinematic Simulations.

## 3 Numerical simulations

### 3.1 Particle trajectories using KS

In this study, the Lagrangian diffusion model Kinematic Simulations (KS) was used to obtain the statistics of particle pair diffusion. In KS one specifies the second order Eulerian structure function through the power spectrum, [14, 21]. KS is useful here because it can generate very large inertial subranges sufficient to test pair diffusion scaling laws.

KS generates turbulent-like non-Markovian particle trajectories by releasing particles in flow fields which are prescribed as sums of energy-weighted random Fourier modes. By construction, the velocity fields are incompressible and the energy is distributed among the different modes by a prescribed Eulerian energy spectrum, *E*(*k*). The essential idea behind KS is that the flow structures in it—eddying, straining, and streaming zones—are similar to those observed in turbulent flows, although not precisely the same, and this is sufficient to generate turbulent-like particle trajectories [14].

KS has been used to examine single particle diffusion [22], and pair diffusion [14, 20, 23–25]. KS has also been used in studies of turbulent diffusion of inertial particles [26–30]. Meneguz & Reeks found that the statistics of the inertial particle segregation in KS generated flow fields for statistically homogeneous isotropic flow fields are similar to those generated by DNS. KS has also been used as a sub-grid scale model for small scale turbulence [31].

### 3.2 The KS velocity fields and energy spectra

An individual Eulerian turbulent flow field realization in KS is generated as a truncated Fourier series,

$$\begin{array}{c} u(x,t)=\sum_{n=1}^{N_{k}}(\left(A_{n}\times {\hat{k}}_{n}\right)\cos\left(k_{n}\cdot x+\omega_{n}t\right)+\left(B_{n}\times {\hat{k}}_{n}\right)\sin\left(k_{n}\cdot x+\omega_{n}t\right)) \end{array}$$ (26)

where *N*_*k*_ is the number of representative wavenumbers, typically hundreds for very long spectral ranges, *R*_*k*_ ≫ 1. ${\hat{k}}_{n}$ is a random unit vector; $k_{n}=k_{n}{\hat{k}}_{n}$ and $k_{n}=\left|k_{n}\right|$. The coefficients **A**_*n*_ and **B**_*n*_ are chosen such that their orientations are randomly distributed in space and uncorrelated with any other Fourier coefficient or wavenumber, and their amplitudes are determined by $\langle A_{n}^{2}\rangle =\langle B_{n}^{2}\rangle \propto E\left(k_{n}\right)dk_{n}$, where *E*(*k*) is the energy spectrum in some wavenumber range *k*_1_ ≤ *k* ≤ *k*_*η*_. The angled brackets 〈⋅〉 denotes the ensemble average over space and over many random flow fields. The associated frequencies are proportional to the eddy-turnover frequencies, $\omega_{n}=\lambda \sqrt{k_{n}^{3}E\left(k_{n}\right)}$. There is some freedom in the choice of λ, so long as 0 ≤ λ < 1. The construction in Eq (26) ensures that the Fourier coefficients are normal to their wavevector which automatically ensures incompressibility of each flow realization, ∇ ⋅ *u* = 0. The flow field ensemble generated in this manner is statistically homogeneous, isotropic, and stationary.

Unlike some other Lagrangian methods, by generating entire kinematic flow fields in which particles are tracked KS does not suffer from the crossing-trajectories error which is caused when two fluid particles occupy the same location at the same time in violation of incompressibility; but because KS flow fields are incompressible by construction this error is eliminated.

The flow at a point in a KS flow field is irregular because of the presence of flow structures such as vortex tubes and a probe will experience irregular alternations between high levels of fluctuations and low levels of fluctuations. However, there is no dynamical energy transfer between different scales of motion so this type of ‘intermittency’ is at a formal level different to real turbulence, [14]. Nevertheless, it is of considerable interest to investigate how Lagrangian statistical scalings change as we adjust the energy spectrum *E*(*k*) ∼ *k*^−*p*^ such that it mimics intermittent-like spectra with *p* ≠ 5/3. This is especially important because KS pair diffusion statistics, including the flatness factor, have been found to be in close agreement with DNS at low Reynolds numbers [25].

The energy spectrum *E*(*k*) can be chosen freely within a finite range of scales, even a piecewise continuous spectrum, or an isolated single mode are possible. To incorporate the effect of large scale sweeping of the inertial scales by the energy containing scales, the simulations are carried out in the swept frame of reference by setting *E*(*k*) = 0 in the largest scales, for *k* < *k*_1_, and an inverse power spectrum in the inertial subrange as discussed in [32],

$$\begin{array}{c} E\left(k\right)=C_{k}\varepsilon^{2/3}L^{5/3-p}k^{-p},\;k_{1}\leq k\leq k_{\eta },\;1<p\leq 3 \end{array}$$ (27)

where *C*_*k*_ is a constant. The Kolmogorov micro-scale is *η* = 2*π*/*k*_*η*_. *L* is some large outer length scale (such as the integral length scale, or a Taylor length scale). The inertial subranges sizes is defined by Eq (1).

A particle trajectory, **x**_*L*_(*t*), is obtained by integrating the Lagrangian velocity, **u**_*L*_(*t*), in time,

$$\begin{array}{ccc} \frac{dx_{L}}{dt} & = & u_{L}\left(t\right)=u\left(x_{L},t\right). \end{array}$$ (28)

Pairs of trajectories are harvested from a large ensemble of flow realizations and pair statistics are then obtained from it for analysis.

The spectrum in (27) is normalized such that the total energy density contained in any flow realization is 3*u*′/2, where *u*′ is the rms turbulent velocity fluctuations in each direction.

In the current simulations, *k*_1_ = 1, *L* = 1, *C*_*k*_ = 1.5 (Kolmogorov constant) and *u*′ = 1. Then this yields,

$$\begin{array}{ccc} \varepsilon^{2/3} & = & \left(p-1\right){\left(1-{\left(\frac{k_{1}}{k_{\eta }}\right)}^{p-1}\right)}^{-1} \end{array}$$ (29)

*p* = 1 is a singular limit which is not consider here. With Eq (29), *v*_*η*_ = (*εη*)^1/3^ is the velocity micro-scale, and *t*_*η*_ = *ε*^−1/3^*η*^2/3^ is the time micro-scale.

The distribution of the wavenumbers is geometric, *k*_*n*_ = *r*^*n*−1^ *k*_1_, and the common ratio is *r* = (*k*_*η*_/*k*_1_)^1/(*N*_*k*_−1)^. The increment in wavenumber-space of the n’th wavenumber is $\Delta k_{n}=k_{n}\left(\sqrt{r}-1/\sqrt{r}\right)$. The frequency factor is chosen to be, λ = 0.5, which is typical in many KS studies. A choice of λ < 1 does not affect the scaling in the pair diffusivity, even frozen turbulence, λ = 0, has been found to yield the same scaling, which has been attributed to the open streamline topology of streamlines in 3D flows, [20].

The particle trajectories *x*_*L*_(*t*) were obtained by integrating Eq (28) using a 4th order Adams-Bashforth predictor-corrector method (4th order Runga-Kutta gives identical results). Thomson & Devenish [33] used a variable time step Δ*t* that was small compared to the turnover time of an eddy of the size of the instantaneous pair separation, but larger than the turbulence micro-scale *t*_*η*_. While this speeds up the turnaround time of the calculations, here we want to avoid any extra assumptions so that unambiguous conclusions can be drawn from the results. Therefore, in all of the current simulations a very small but fixed time step, Δ*t* ≪ *t*_*η*_ has been used. This has the further advantage of avoiding any smoothening of the particle trajectories that is necessary when using variable time steps.

Eight pairs of a particles were released in each flow realization, placed far enough apart for each pair to be independent. It is crucial to run over a large number of different flow realizations, otherwise the statistics will not converge. Typically the ensemble was around 4000 flow realizations, yielding a harvest of 32, 000 particle pair trajectories per simulation. A simulation was run for about one large timescale, *T* = 2*π*/*k*_1_, which required around 10^6^ time steps for *R*_*k*_ = 10^6^, and about 10^3^ time steps for *R*_*k*_ = 10^1^. In most of the simulations the initial separation was *l*_0_ ≈ *η*; but for *p* → 3 the energy in the small scales even in the inertial subrange is so small that the particles take a long time to move apart significantly, so in order to accelerate the simulations for these cases we took *l*_0_ ≫ *η*.

## 4 Simulation results

### 4.1 Infinite inertial subrange *R*_*k*_ → ∞ (*Re* → ∞)

In this first set of simulations we investigate the theory for pair diffusion in asymptotically infinite inertial subrange, (infinite Reynolds number), and for this we take *R*_*k*_ = 10^6^.

The spectra in Eq (27) were taken as input to the KS simulations. It is important to simulate cases over the whole range of energy spectra 1 < *p* ≤ 3, in order to examine the new theory comprehensively; 25 cases of *p* were selected in the range, 1 < *p* ≤ 3. The case *p* = 1 is singular, but *p* can be taken close to this limit; the smallest value of *p* chosen was, 1.01 (Table 1).

**Table 1:** *p*, *γ*(*p*), *γ*^*l*^(*p*), *χ*(*p*), *χ*^*l*^(*p*), and *M*_*γ*_(*p*) from the simulations for *R*_*k*_ = 10^6^. The pair diffusion coefficient is, $K∼\sigma_{l}^{\gamma (p)}$, and the locality scaling is, *γ*^*l*^(*p*) = (1 + *p*)/2. The mean square separation is, 〈*l*^2^〉 = *t*^*χ*(*p*)^, where *χ*(*p*) = 2/(2 − *γ*(*p*)), and the locality scaling is, *χ*^*l*^(*p*) = 2/(2 − *γ*^*l*^(*p*)). The ratio of the power scalings is, *M*_*γ*_(*p*) = *γ*(*p*)/*γ*^*l*^(*p*).

*p*	*γ*(*p*)	*γ*^*l*^(*p*)	*χ*(*p*)	*χ*^*l*^(*p*)	*M*_*γ*_
1.01	1.060	1.005	2.128	2.010	1.055
1.1	1.120	1.05	2.273	2.105	1.067
1.2	1.190	1.10	2.469	2.222	1.082
1.3	1.260	1.15	2.703	2.353	1.096
1.4	1.340	1.20	3.030	2.500	1.117
1.5	1.410	1.25	3.390	2.667	1.128
1.6	1.480	1.30	3.846	2.857	1.139
5/3	1.525	4/3	4.211	3	1.144
1.70	1.545	1.35	4.396	3.077	1.144
1.72	1.556	1.360	4.505	3.125	1.144
1.74	1.570	1.370	4.651	3.175	1.146
1.77	1.585	1.385	4.819	3.252	1.144
1.8	1.605	1.40	5.063	3.333	1.146
1.9	1.660	1.45	5.882	3.636	1.145
2.0	1.710	1.50	6.897	4	1.140
2.1	1.750	1.55	8.000	4.444	1.129
2.2	1.790	1.60	9.424	5	1.119
2.3	1.820	1.65	11.11	5.714	1.103
2.4	1.850	1.70	13.33	6.676	1.088
2.5	1.880	1.75	16.67	8	1.074
2.6	1.900	1.80	20.00	10	1.056
2.7	1.930	1.85	28.57	13.33	1.043
2.8	1.950	1.90	40.00	20	1.026
2.9	1.970	1.95	67.67	40	1.010
3.0	2.000	2.00	∞	∞	1.000

The results obtained from the simulations for *R*_*k*_ = 10^6^ are summarized in Table 1. This table shows *γ*(*p*) from the simulations in column 2; and the locality scalings *γ*^*l*^(*p*) = (1 + *p*)/2 in column 3. For the mean square separation laws, 〈*l*^2^〉 ∼ *t*^*χ*^, the *χ*(*p*) = 2/(2 − *γ*(*p*)) is shown in column 4; and the locality scalings $\chi^{l}\left(p\right)=2/\left(2-\gamma_{p}^{l}\right)=4/\left(3-p\right)$ in column 5. The ratio of scaling powers,

$$\begin{array}{ccc} M_{\gamma }\left(p\right) & = & \frac{\gamma (p)}{\gamma^{l}\left(p\right)}, \end{array}$$ (30)

is shown in column 6.

**Fig 2:**
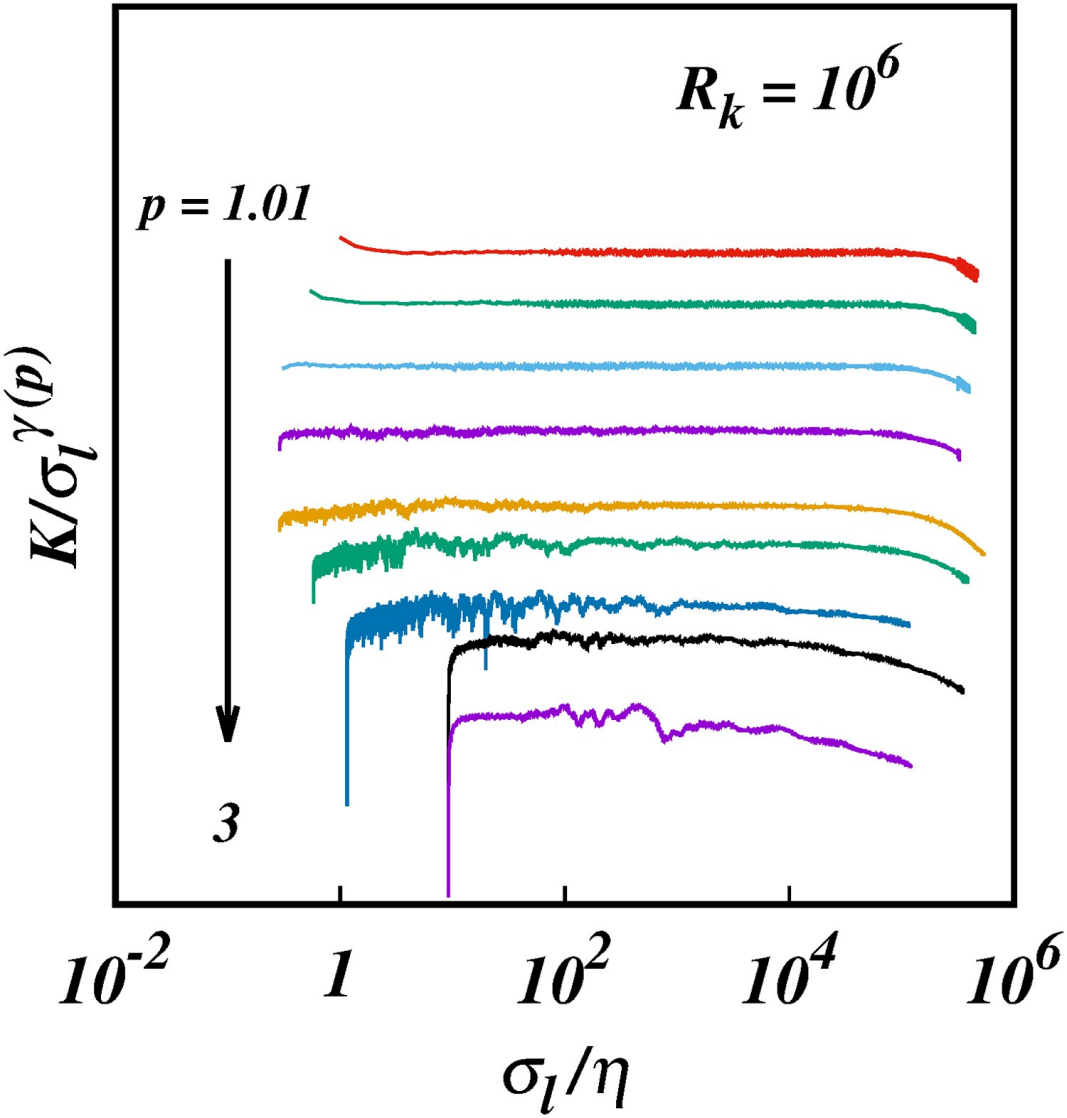
The compensated turbulent pair diffusion coefficient, $\log(K/\sigma_{l}^{\gamma (p)})$, against log(*σ*_*l*_/*η*), for the same cases as in Fig 1, and with the same colour coding. For clarity some of the plots have been spaced out vertically, hence no scale is shown on the ordinate; this does not affect the scalings.

Log-log plots of the turbulent pair diffusion coefficient *K*/*ηv*_*η*_ against the separation *σ*_*l*_/*η* for different energy spectra *E*(*k*) ∼ *k*^−*p*^ display clear power law scalings of the form,

$$\begin{array}{ccc} K(l,p) & ∼ & \sigma_{l}^{\gamma (p)},\;1<\gamma \left(p\right)\leq 2,\;1<p\leq 3, \end{array}$$ (31)

over wide ranges of the separation inside the inertial subrange, Fig 1. The *γ*(*p*)’s are the slopes of the plots in Fig 1; these are more easily observed by plotting the compensated diffusion coefficient $K/\sigma_{l}^{\gamma (p)}$ against the separation, Fig 2. The *γ*(*p*)’s are obtained as the powers that give horizontal lines over the longest range of separation, a procedure that determines *γ*(*p*) to within 1% error for most *p*, except near the singular limit, *p* = 1, where the errors are around 6%, (see Section 4).

**Fig 3:**
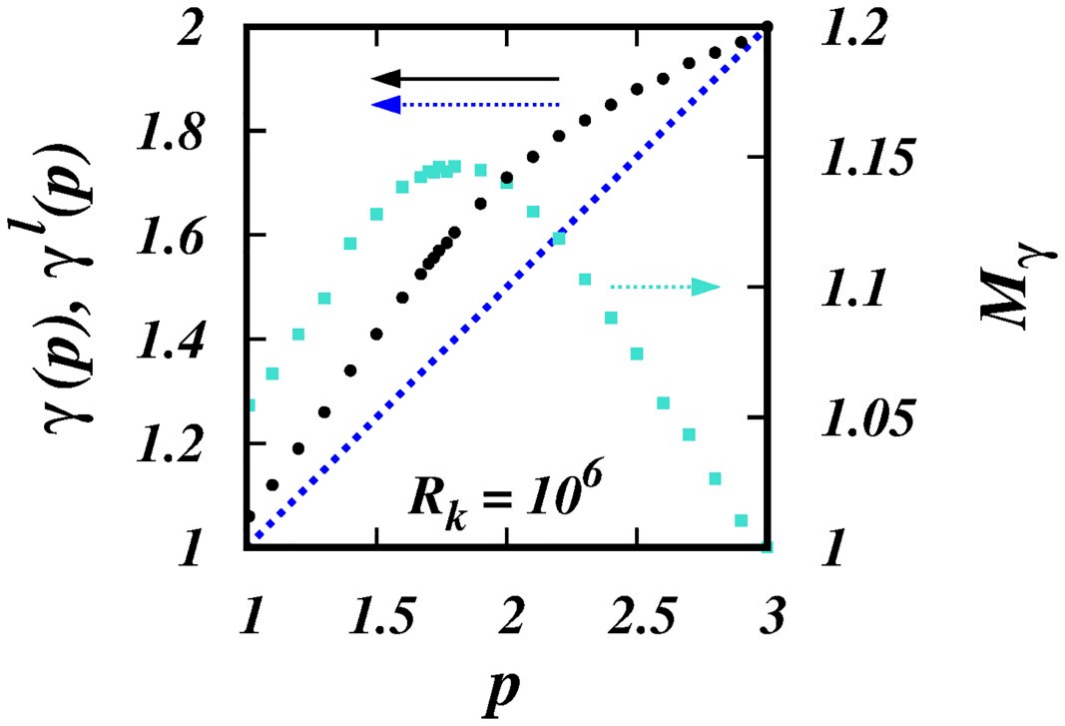
*γ*(*p*), *γ*^*l*^(*p*), and *M*_*γ*_(*p*) against *p*. The black filled circles are the *γ*(*p*)’s from the simulations. The dotted blue line is the locality scaling *γ*^*l*^(*p*) = (1 + *p*)/2. The cyan squares are the ratios, *M*_*γ*_ = *γ*(*p*)/*γ*^*l*^(*p*) (right hand scale). See Table 1.

Fig 3 shows the plots of *γ*(*p*) (black filled circles) and *γ*^*l*^(*p*) = (1 + *p*)/2 (dotted blue line) against *p*. It is observed that the scaling powers, *γ*(*p*), are in the range, (1 + *p*)/2 < *γ*(*p*) ≤ 2, and as *p* → 1, then *γ*(*p*) → 1; and as *p* → 3, then *γ*(*p*) → 2. Furthermore, the, *γ*(*p*)’s, display a smooth transition between the asymptotic limits at *p* = 1 and 3.

The range of separation over which the scalings laws, $K\left(l,p\right)∼\sigma_{l}^{\gamma (p)}$, exist progressively reduce from both ends as *p* → 3. Ultra-violet corrections reduces the range from below due to the diminishing energies contained in the smallest scales so that the pair separation penetrates further into the inertial subrange before it ‘forgets’ the initial separation. Infra-red corrections reduces the range from above because the long range correlations are increasingly stronger as *p* → 3 causing the particles in a pair to become independent at earlier times and at smaller separation.

The plot of *M*_*γ*_ against *p* in Fig 3 (right hand scale) shows a peak at *p*_*m*_ ≈ 1.8, where *M*_*γ*_(*p*_*m*_) ≈ 1.15. *M*_*γ*_ remains close to this peak value in a neighbourhood of *p*_*m*_, in the range 1.5 < *p* < 2.

**Fig 4:**
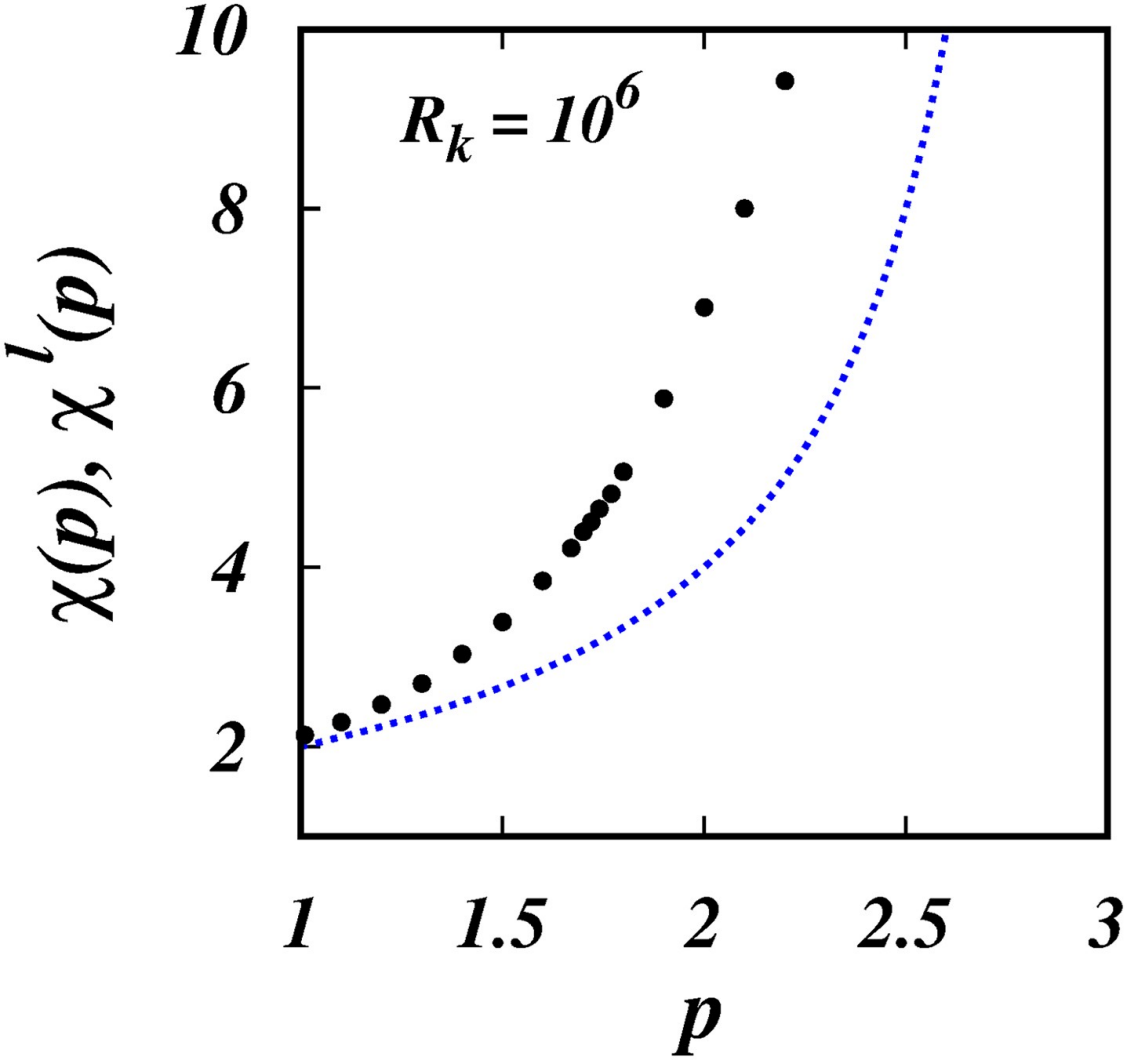
*χ*(*p*), *χ*^*l*^(*p*), against *p*. The black filled circles are, *χ*(*p*)’s from the simulations. The dotted blue line is the locality scaling, *χ*^*l*^(*p*) = 4/(3 − *p*). See Table 1.

**Fig 5:**
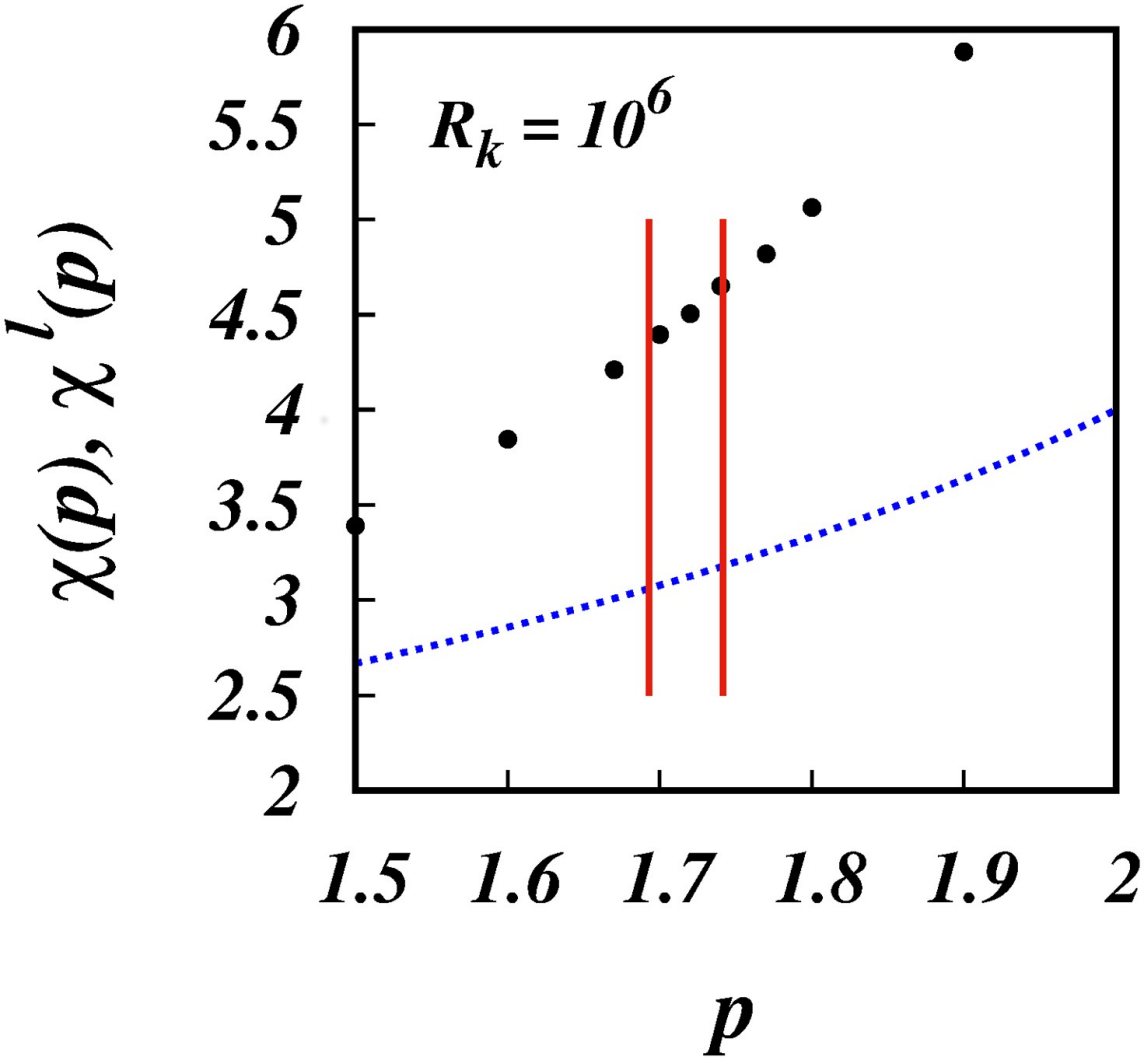
*χ*(*p*), *χ*^*l*^(*p*), against *p*. Similar to Fig 4, but focussing in on the range of intermittency spectra. The two vertical red lines are the lower and upper bounds for intermittent turbulence spectra, respectively *p* = 1.70 and 1.74.

Fig 4 shows the simulation results of the scaling powers, *χ*(*p*), (filled black circles), and *χ*^*l*^(*p*) = 4/(3 − *p*) (dotted blue line), against *p*. The same is shown in Fig 5, but focussing in on the range 1.5 < *p* < 2 which covers the intermittent turbulence range which is indicated by the two red vertical lines.

Turbulence with intermittency has spectrum *E*(*k*) ∼ *k*^−(5/3 + *μ*_*I*_)^. Three extra cases with, *p*_*I*_ = 5/3+*μ*_*I*_, where therefore simulated. The currently accepted value for the intermittency lies in the range, 0.025 < *μ*_*I*_ < 0.075, [34–37]; and three cases that cover this range are, *p*_*I*_ = 1.70, 1.72, and 1.74. For these spectra, the simulations produced, (Fig 3 and Table 1),

$$\begin{array}{ccc} K_{\mu_{I}} & ∼\sigma_{l}^{1.545}, & \;\text{for}\;p_{I}=1.70,\;\;\mu_{I}=0.033\\ K_{\mu_{I}} & ∼\sigma_{l}^{1.556}, & \;\text{for}\;p_{I}=1.72,\;\;\mu_{I}=0.053\\ K_{\mu_{I}} & ∼\sigma_{l}^{1.570}, & \;\text{for}\;p_{I}=1.74,\;\;\mu_{I}=0.073 \end{array}$$ (32)

The mid-point in the intermittency range is close to, *p*_*I*_ = 1.72, which gives the scaling law $K_{\mu_{I}}∼\sigma_{l}^{1.556}$, which is an error of about 0.5% in the scaling power compared to the Richardson’s 1926 revised data as shown in Fig 1 in [12]. For, *p*_*I*_ = 1.70, we obtain $K_{\mu_{I}}∼\sigma_{l}^{1.545}$, and the error in the scaling power is 1.2%; for *p*_*I*_ = 1.74, we obtain $K_{\mu_{I}}∼\sigma_{l}^{1.570}$, and the error in the scaling power is 0.4%.

The corresponding scalings for the mean square separation, 〈*l*^2^〉 ∼ *t*^*χ*(*p*)^, for the three intermittent spectra are respectively, ∼ *t*^4.396^, ∼ *t*^4.505^ and ∼ *t*^4.651^, Fig 5 and Table 1.

The Kolmogorov spectrum *p* = 5/3 produces, $K∼\sigma_{l}^{1.525}$, and the error in the scaling power is 2.5%. Richardson’s 4/3-law, $K∼\sigma_{l}^{4/3}$, is 15% in error.

The simulations show a non-Richardson 4/3-scaling, $K∼\sigma_{l}^{4/3}$, for the the spectrum *E*(*k*) ∼ *k*^−1.4^, where p_*_ ≈ 1.4, (Fig 3 and Table 1); this yields a new non-R-O *t*^3^-regime, 〈*l*^2^〉 ∼ *t*^3^.

### 4.2 Short inertial subrange *R*_*k*_ ≪ ∞ (*Re* ≪ ∞)

In this section, we investigate the new theory in [12] in the limit of short inertial subrange. According to the new theory short subranges could isolate and expose the local diffusional process.

Simulations were carried out for the same cases of *p* as in Section 3.1, but now for a short inertial subrange of *R*_*K*_ = 10^2^. The results are summarised in Table 2 which shows *p*, *γ*(*p*), *χ*(*p*), and *M*_*γ*_(*p*). Figs 6 to 10 are the counterparts to Figs 1 to 5 but for *R*_*k*_ = 10^2^.

**Table 2:** *p*, *γ*(*p*), *χ*(*p*), and *M*_*γ*_(*p*) from the simulations for *R*_*k*_ = 10^2^. The pair diffusion coefficient is, $K∼\sigma_{l}^{\gamma (p)}$. The mean square separation is, 〈*l*^2^〉 = *t*^*χ*(*p*)^, where *χ*(*p*) = 2/(2 − *γ*(*p*)). The ratio of the power scalings is, *M*_*γ*_(*p*) = *γ*(*p*)/*γ*^*l*^(*p*).

*p*	*γ*(*p*)	*χ*(*p*)	*M*_*γ*_
1.01	1.100	2.222	1.095
1.1	1.150	2.353	1.095
1.2	1.210	2.532	1.100
1.3	1.250	2.667	1.087
1.4	1.305	2.878	1.088
1.5	1.350	3.077	1.080
1.6	1.390	3.279	1.069
5/3	1.410	3.390	1.057
1.70	1.420	3.449	1.052
1.72	1.430	3.509	1.051
1.74	1.440	3.571	1.051
1.77	1.455	3.670	1.051
1.8	1.465	3.738	1.046
1.9	1.510	4.082	1.041
2.0	1.540	4.348	1.027
2.1	1.565	4.600	1.010
2.2	1.610	5.128	0.006
2.3	1.640	5.555	0.994
2.4	1.670	6.061	0.982
2.5	1.700	6.667	0.971
2.6	1.750	8.000	0.972
2.7	1.800	10.00	0.973
2.8	1.860	14.29	0.979
2.9	1.930	28.57	0.990
3.0	2.000	∞	1.000

**Fig 6:**
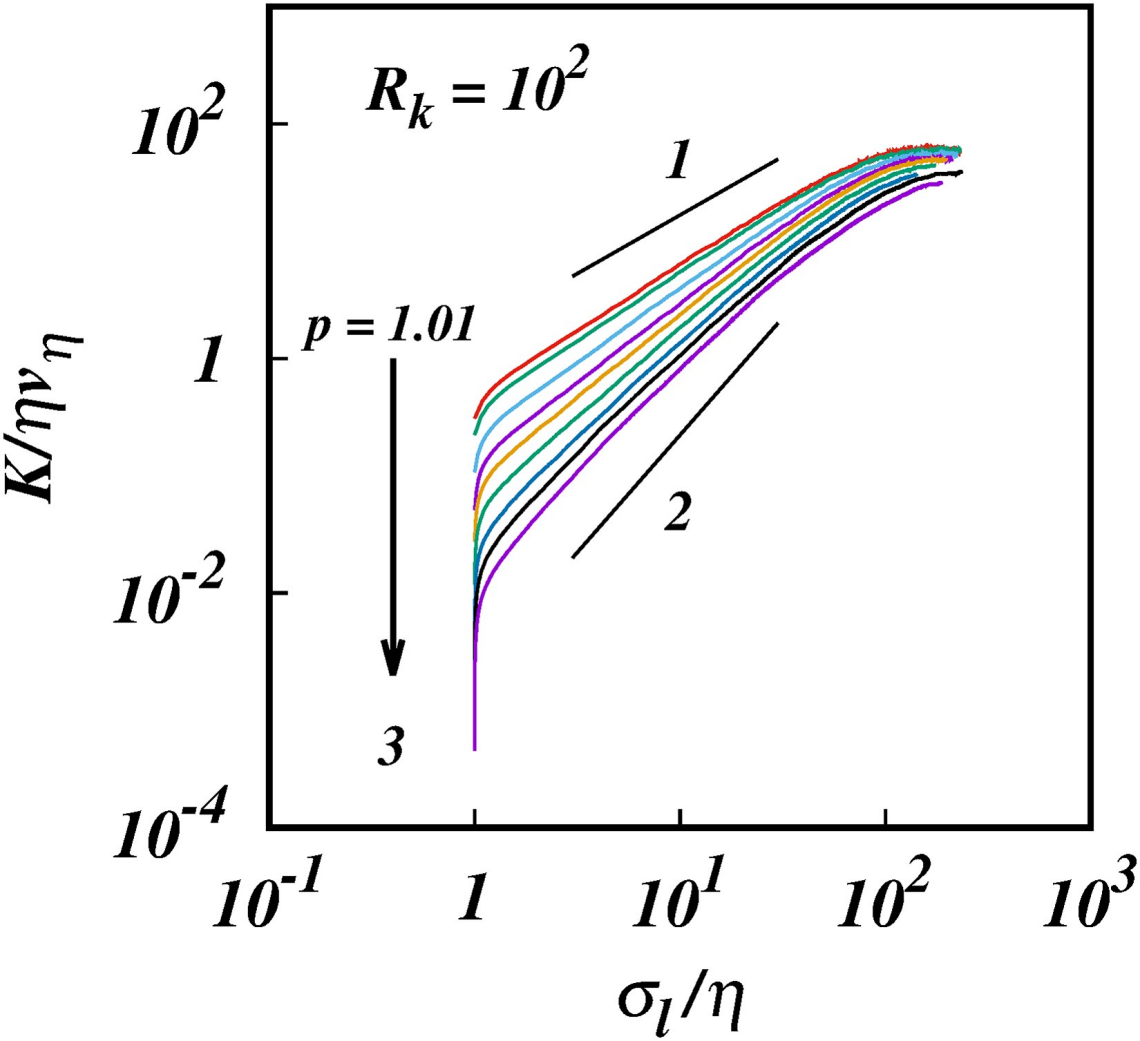
The turbulent pair diffusion coefficient, log(*K*/*ηv*_*η*_), against, log(*σ*_*l*_/*η*), from simulations with energy spectra, *E*(*k*) ∼ *k*^−*p*^, and inertial subrange size, *R*_*k*_ = 10^2^. For clarity only 10 of the 25 cases in Table 2 are shown: *p* = 1.01, 1.1,1.3, 1.5, 5/3,1.9,2.2,2.5, 2.9,3.0. Solid black lines of slopes 1 and 2 are shown for comparison.

**Fig 7:**
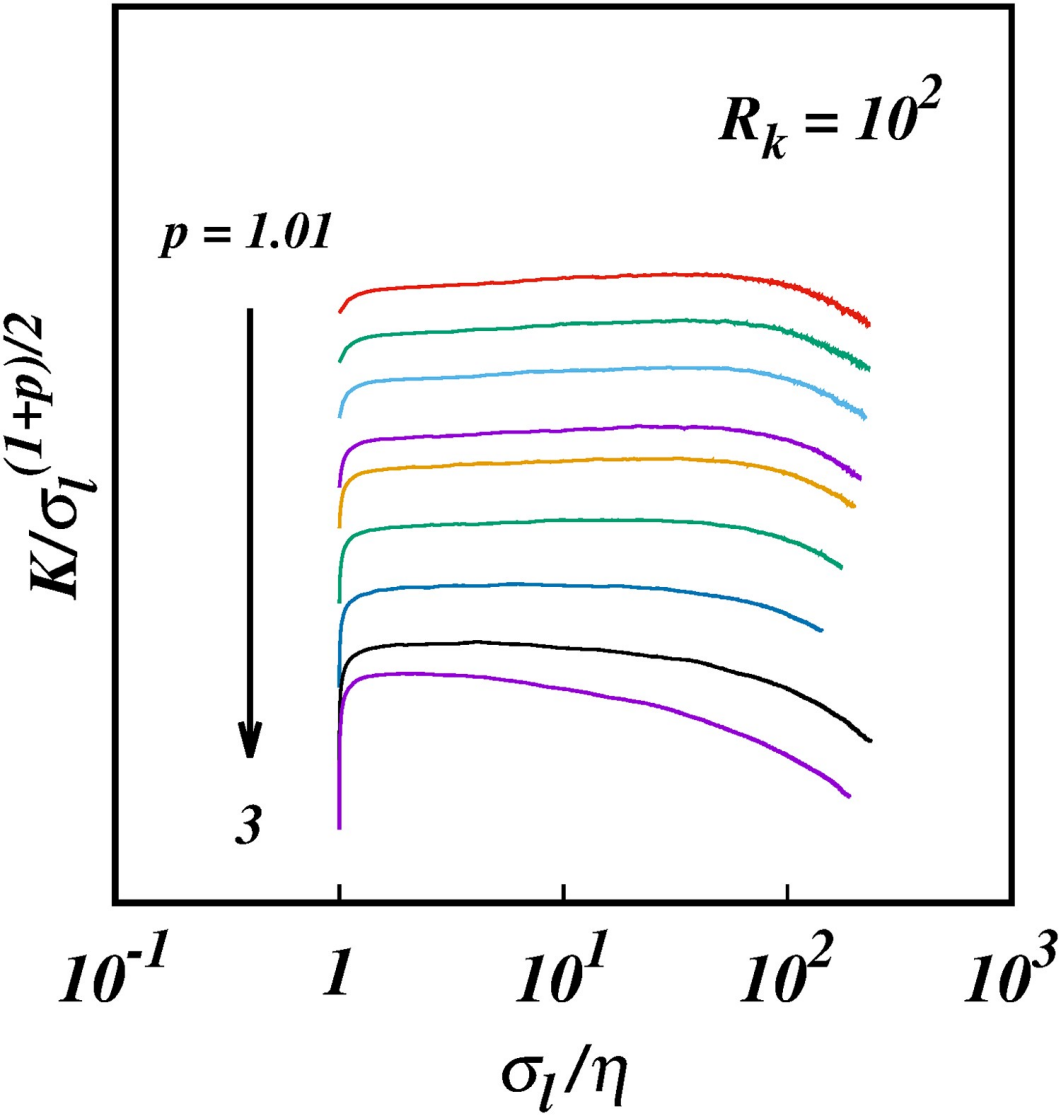
The compensated turbulent pair diffusion coefficient, $\log(K/\sigma_{l}^{\gamma (p)})$, against log(*σ*_*l*_/*η*), for *R*_*k*_ = 10^2^ for the same cases as in Fig 6, and with the same colour coding. For clarity some of the plots have been spaced out vertically, hence no scale is shown on the ordinate; this does not affect the scalings.

**Fig 8:**
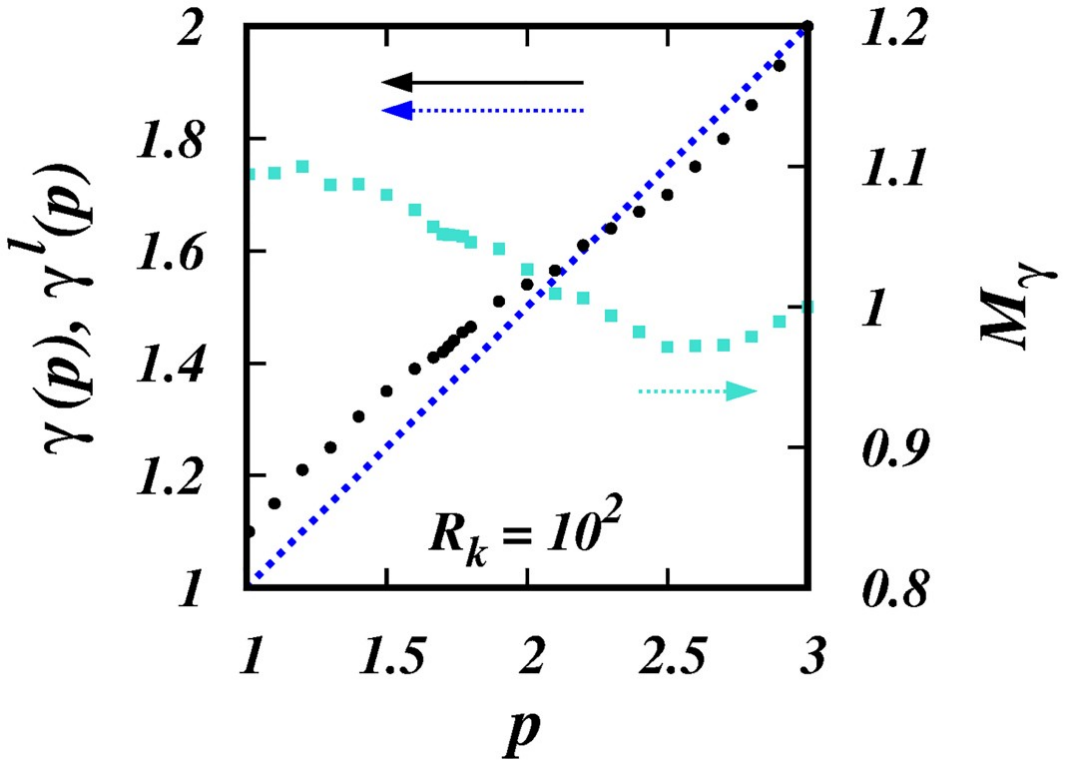
*γ*(*p*), *γ*^*l*^(*p*), and *M*_*γ*_(*p*) against *p* for *R*_*k*_ = 10^2^. The black filled circles are the *γ*(*p*)’s from the simulations. The dotted blue line is the locality scaling *γ*^*l*^(*p*) = (1 + *p*)/2. The cyan squares are the ratios, *M*_*γ*_ = *γ*(*p*)/*γ*^*l*^(*p*) (right hand scale). See Table 2.

**Fig 9:**
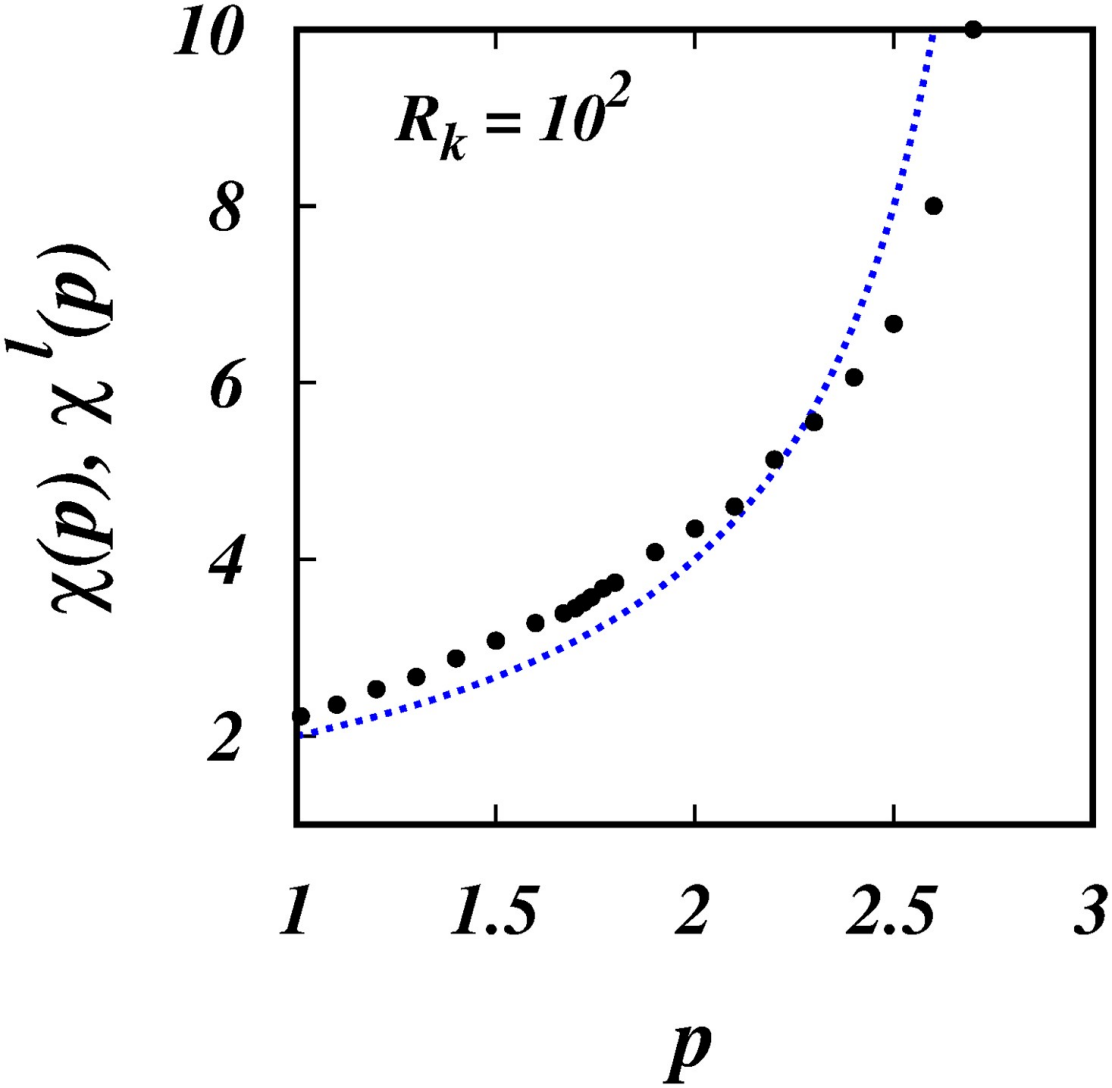
*χ*(*p*), *χ*^*l*^(*p*), against *p* for *R*_*k*_ = 10^2^. The black filled circles are, *χ*(*p*)’s from the simulations. The dotted blue line is the locality scaling, *χ*^*l*^(*p*) = 4/(3 − *p*). See Table 2.

**Fig 10:**
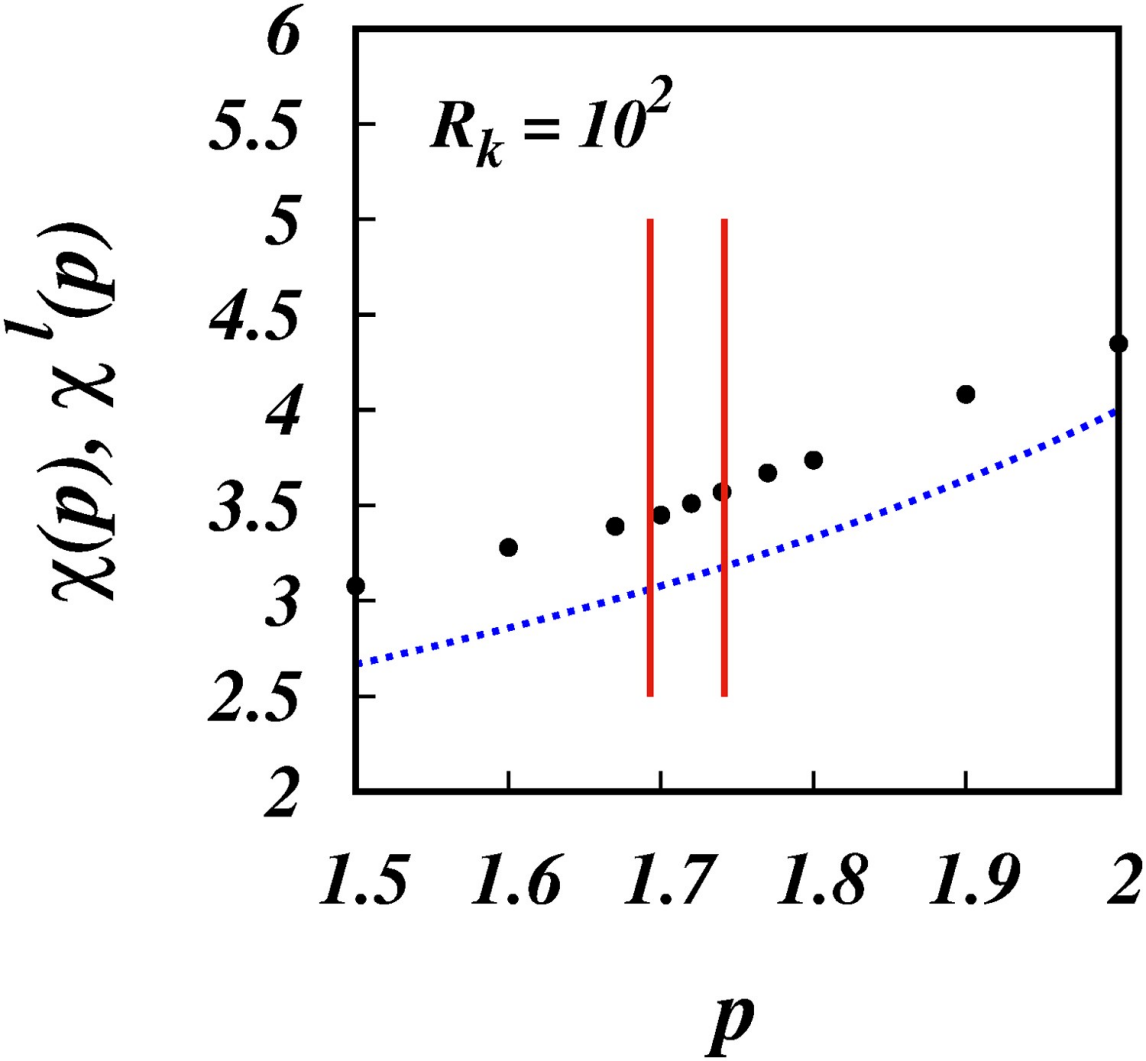
*χ*(*p*), *χ*^*l*^(*p*), against *p* for *R*_*k*_ = 10^2^. Similar to Fig 8, but focussing in the range of real turbulence with intermittency. The two vertical red lines are the lower and upper bounds for intermittent turbulence spectra, respectively *p* = 1.70 and 1.74.

Fig 6 shows the log-log plots of the diffusion coefficient *K*/*ηv*_*η*_ against *σ*_*l*_/*η* for the same 10 selected cases of *p* as in Fig 1. Fig 7 shows the corresponding log-log plots of the compensated diffusion coefficient, $K/\sigma_{l}^{(1+p)/2}$ against *σ*_*l*_/*η*—this time the diffusion coefficient is compensated by the locality scaling $∼\sigma_{l}^{(1+p)/2}$. The plots are close to horizontal, though not quite.

Table 2 shows the *γ*(*p*)’s obtained from the simulations, which are also plotted in Fig 8 (c.f. Fig 3). The *γ*(*p*)’s are close to the locality scalings *γ*_*l*_(*p*) = (1 + *p*)/2, as demostrated in the plot of the ratio, *M*_*γ*_ against *p* also in Fig 8. Although they differ by about 10% close to *p* = 1, most of this error is due to the singularity at this point, see Section 4.

In the critical range of *p* close to *p* = 5/3, which is far from the singular limit, the maximum difference from locality is about 5% which is a real physical effect most likely due to the fact that even in this short subrange there may be some small non-local straining effect. There is also infra-red corrections close to *k* = *k*_1_, and ultra-violet corrections close to *k* = *k*_*η*_ which will penetrate some way in to the inertial subrange from both ends thus reducing the effective range over which the scalings can be observed. In short inertial subranges these end-of-range corrections will be appear relatively greater than in large inertial subranges.

As in the latter case, as *p* → 3, the range of separations over which the locality scaling is observed diminishes from both ends of the domain, and this probably leads to the sub-local diffusion, *γ*(*p*) < *γ*^*l*^(*p*), for *p* > 2.2, Fig 8.

Fig 9 shows the plots of *χ*(*p*) and the locality scalings *χ*^*l*^(*p*) against *p* for comparison, (c.f. Fig (4)). Fig 10 is the same except zooming in on the intermittency range (c.f. Fig 5).

From the simulations, the scalings obtained for the pair diffusivity are: $K∼\sigma_{l}^{1.41}$ for *p* = 5/3; $K∼\sigma_{l}^{1.42}$ for *p* = 1.70; $K∼\sigma_{l}^{1.43}$ for *p* = 1.72; $K∼\sigma_{l}^{1.44}$ for *p* = 1.74.

For the same spectra, the scalings for 〈*l*^2^〉 are respectively, ∼ *t*^3.39^ ∼ *t*^3.449^ ∼ *t*^3.509^ and ∼ *t*^3.571^, Fig 10 and Table 2. Thus, a true R-O *t*^3^-regime cannot exist in reality.

Overall, the simulation results for *R*_*k*_ = 10^2^ are always close to locality, and to within 5% in the range of spectra close to real turbulent spectra—we call these quasi-local regimes.

### 4.3 Scaling law as *R*_*k*_ increases

The theory in [12] predicts a smooth transition from local to non-local regimes as *R*_*k*_ increases. To examine this, simulations were carried out for progressively larger inertial subranges, from *R*_*k*_ = 10^1^ to 10^6^, for two selected energy spectra, namely for Kolmogorov spectrum, *E*(*k*) ∼ *k*^−5/3^, and for an intermittent spectrum, *E*(*k*) ∼ *k*^−1.72^.

**Fig 11:**
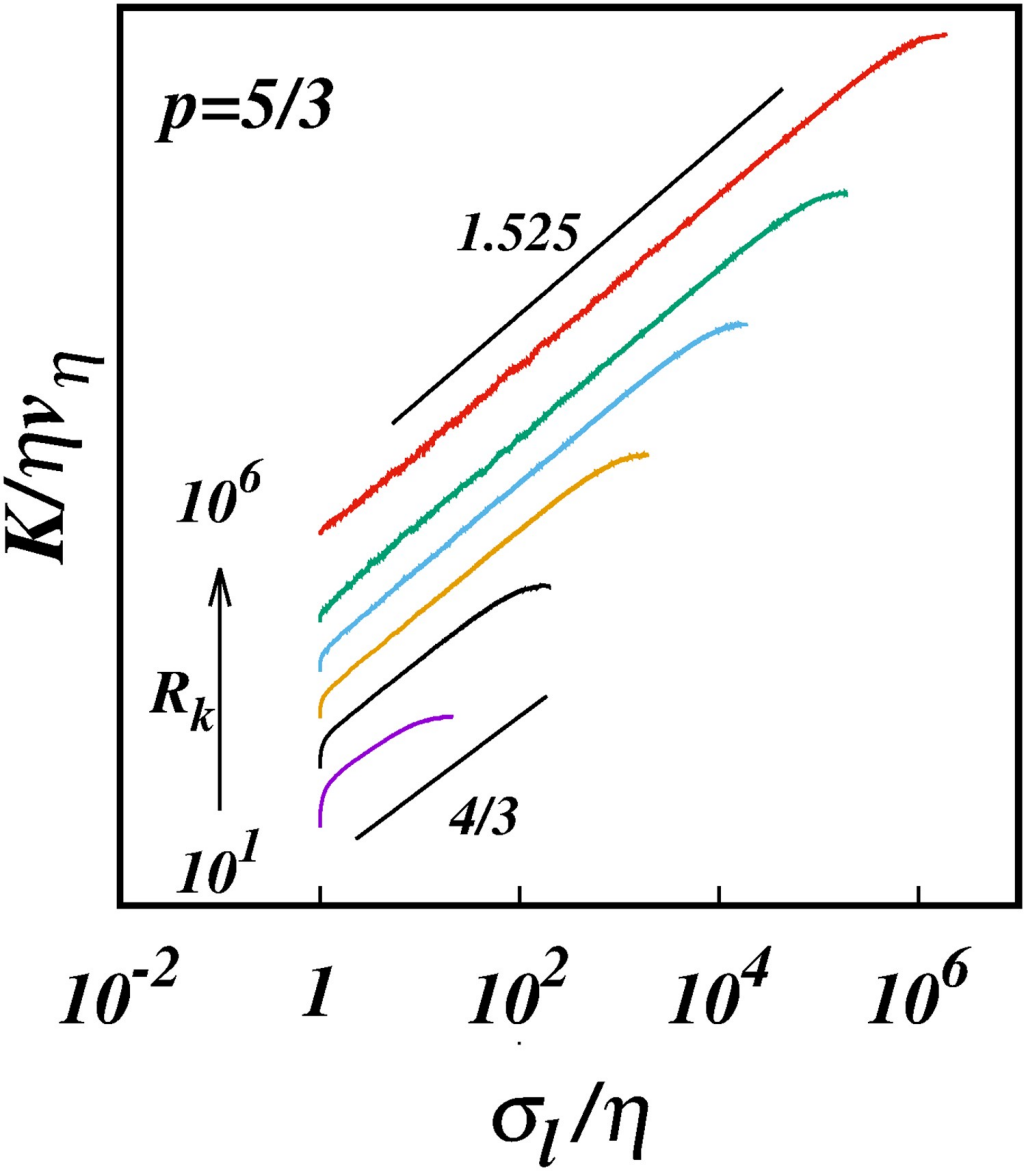
The turbulent pair diffusion coefficient, log(*K*/*ηv*_*η*_), against, log(*σ*_*l*_/*η*), from the simulations for spectrum *E*(*k*) ∼ *k*^−5/3^ for different inertial ranges *R*_*k*_ as shown in Table 3. Lines of slope 4/3 and 1.525 are shown for comparison.

**Fig 12:**
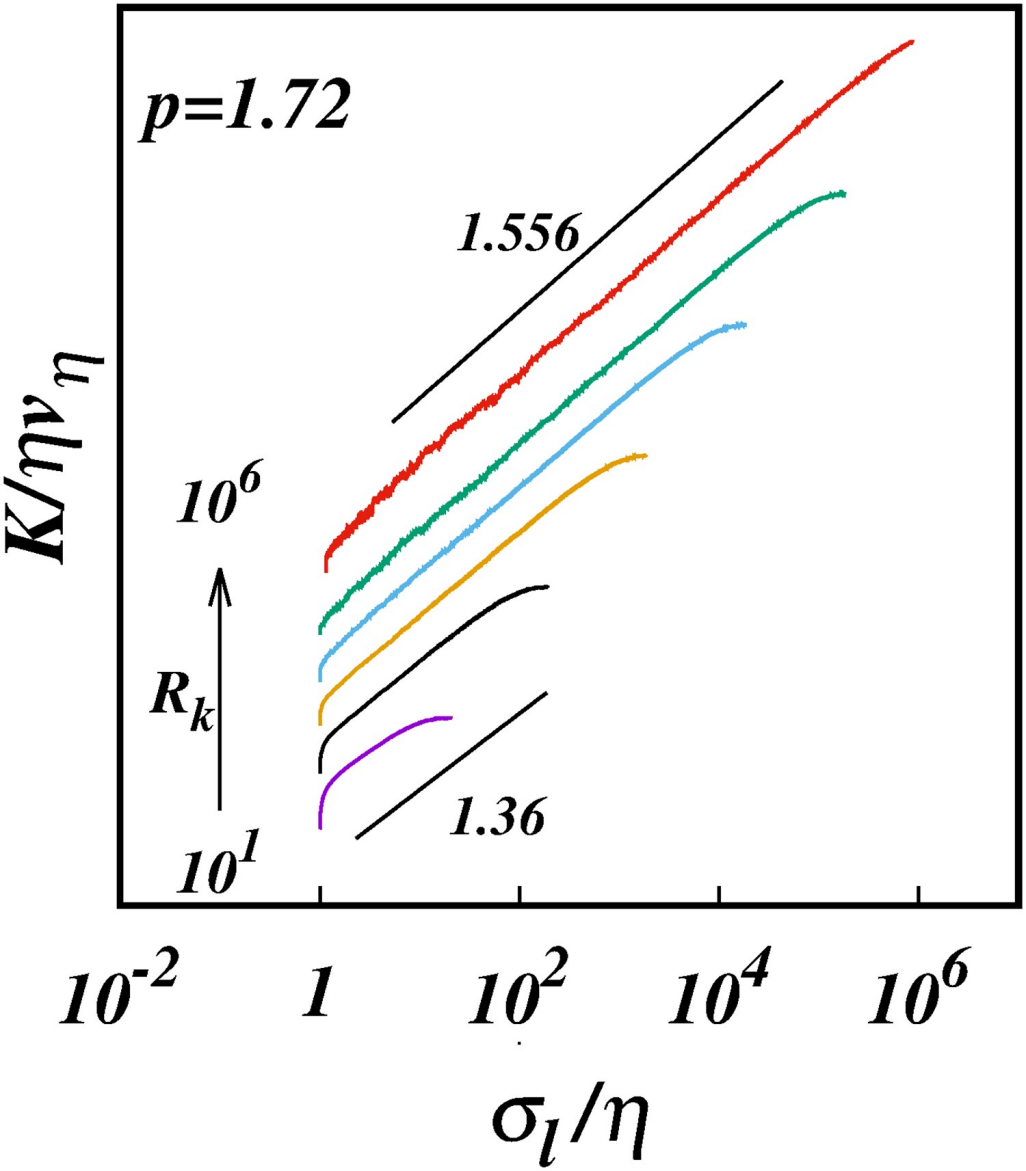
The turbulent pair diffusion coefficient, log(*K*/*ηv*_*η*_), against, log(*σ*_*l*_/*η*), from the simulations for spectrum *E*(*k*) ∼ *k*^−1.72^ for different inertial ranges *R*_*k*_ as shown in Table 3. Lines of slope 1.36 and 1.556 are shown for comparison.

Fig 11 shows the log-log plots of the diffusion coefficient *K*/*ηv*_*η*_ against *σ*_*l*_/*η* for *p* = 5/3 for the six cases of inertial subrange size considered, Table 3. Fig 12 is similar but for *p* = 1.72. Both sets of plots show similar trends; for the smallest *R*_*k*_ = 10^1^ we obtain *γ* ≈ 1.1 ≪ 4/3—clearly the subrange is too short to manifest any kind of genuine inertial range scaling.

**Table 3:** *R*_*k*_, *γ* and *χ*, from the simulations for *p* = 5/3, and *p* = 1.72 (right hand columns).

	*p* = 5/3		*p* = 1.72	
*R*_*k*_	*γ*(*p*)	*χ*(*p*)	*γ*(*p*)	*χ*(*p*)
10^1^	1.220	2.564	1.230	2.597
10^2^	1.410	3.390	1.440	3.571
10^3^	1.472	3.788	1.495	3.960
10^4^	1.500	4.000	1.530	4.255
10^5^	1.515	4.124	1.550	4.444
10^6^	1.525	4.211	1.556	4.505

The cases *R*_*k*_ = 10^2^ are close to locality scalings, as already discussed in Section 3.2.

**Fig 13:**
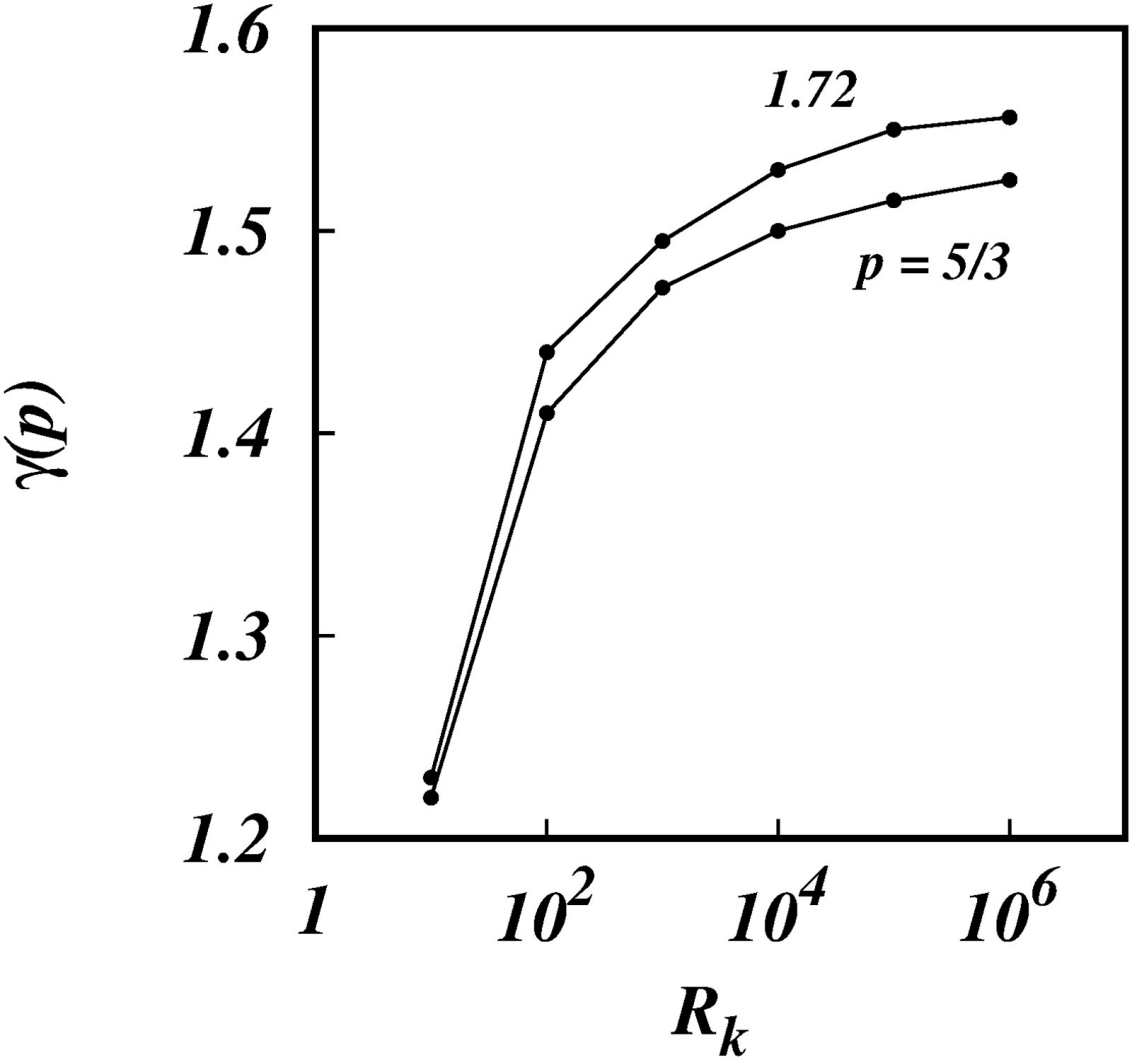
*γ*, against, log(*R*_*k*_), from the simulations (symbols) for spectra *E*(*k*) ∼ *k*^−5/3^ and *k*^−1.72^ as shown.

**Fig 14:**
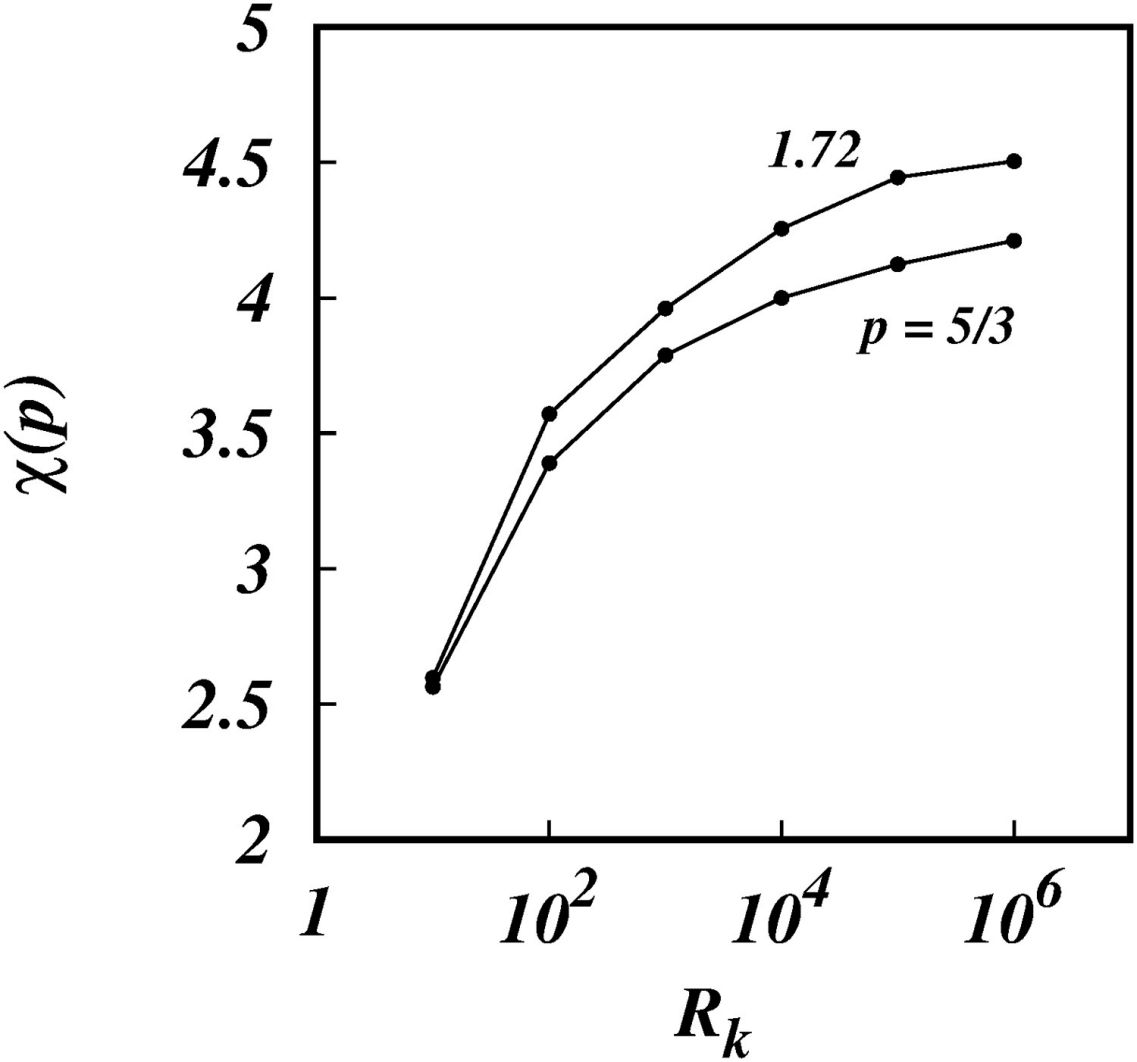
*χ*, against, log(*R*_*k*_), from the simulations (symbols) for spectra *E*(*k*) ∼ *k*^−5/3^ and *k*^−1.72^ as shown.

As *R*_*k*_ increases further the *γ*(*p*)’s increase asymptotically towards the limiting values, *γ* → 1.525 for *p* = 5/3, and *γ* → 1.556 for *p* = 1.72. This is clearly seen in Figs 13 and 14 which show the log-linear plots of *γ* against log(*R*_*k*_).

## 5 Exposing the non-local process

In [12] it was hypothesised that the non-local process could also be isolated and exposed by taking a very small initial separation *l*_0_ ≪ *η*. Then the early motion should be purely strain dominated relative motion so long as *σ*_*l*_(*t*) ≪ *η*. It was also noted that this regime should be independent of the form of *E*(*k*) and also of the size of the inertial subrange. To examine this hypothesis we therefore only need to test a few cases to prove generality.

**Fig 15:**
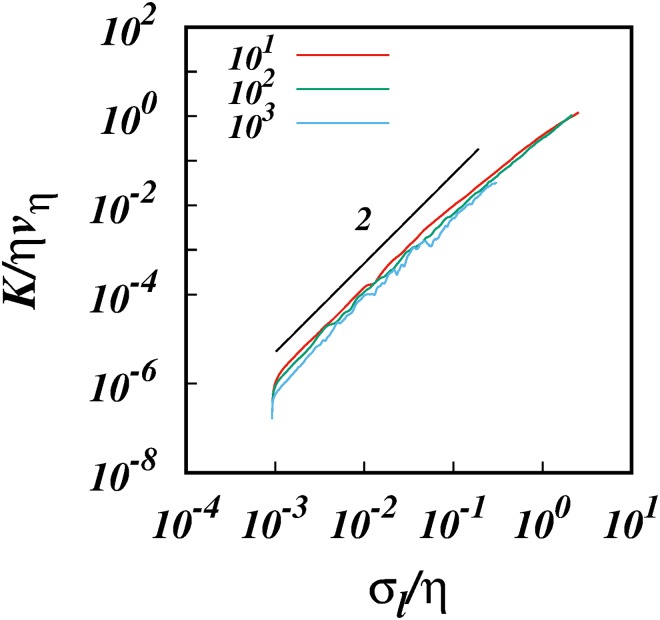
log(*K*/*ηv*_*η*_), against, log(*σ*_*l*_/*η*), from the simulations for the spectrum *E*(*k*) ∼ *k*^−5/3^ for different inertial subrange size, *R*_*k*_ = 10^1^, 10^2^, and 10^3^ as shown. The initial separations in each case is *l*_0_/*η* = 10^−3^. A line of slope 2 is shown for comparison.

**Fig 16:**
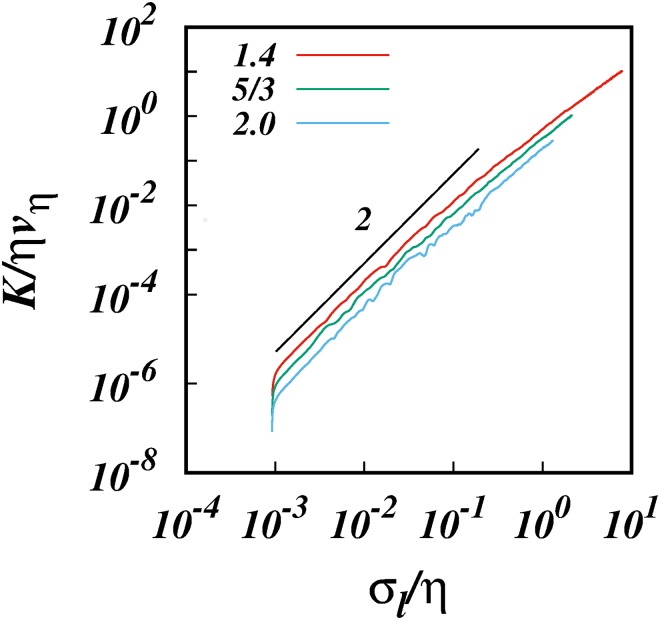
log(*K*/*ηv*_*η*_), against, log(*σ*_*l*_/*η*), from the simulations for *R*_*k*_ = 10^2^ for different spectra *E*(*k*) ∼ *k*^−*p*^, *p* = 1.4, 5/3, and 2.0 as shown. The initial separations in each case is *l*_0_/*η* = 10^−3^. A line of slope 2 is shown for comparison.

Thus we ran simulations for three sizes of the subrange, *R*_*k*_ = 10^1^, 10^2^, and 10^3^ for the same spectrum *p* = 5/3, and in each case we took *l*_0_/*η* = 10^−3^ respectively. Fig 15 shows log-log plot of *K*/*ηv*_*η*_ against *σ*_*l*_/*η* for these cases. We also ran simulations for different spectra *p* = 1.4, 5/3, and 2.0 for the same inertial subrange size *R*_*k*_ = 10^2^, and the results are shown in Fig 16.

In all the above cases, the results display clear $K∼\sigma_{l}^{2}$ scalings which is the signature of strained motion. Importantly, these regimes are valid till about *σ*_*l*_/*η* ≈ 10^−2^ before they start to bend away from from a slope of 2 as the inertial subrange is approached from below and inertial subrange scaling begins to have its impact. This gives us a rough estimate of the size of the locality kernel which must be of the order of *C* ≈ 10^2^.

## 6 Estimate of numerical errors

The numerical results presented here are the most comprehensive obtained to date from KS due to the very large ensemble of particle pairs and the small time steps used. The statistical fluctuations in the results are therefore small. The *γ*(*p*)’s, which are the slopes of the plots in Fig 5, can be determined to within 1% error. An exception is close to the singular limit *p* = 1 where the numerical errors can be large. An accurate estimate of this error can be obtained as follows, noting first that the error level in *γ*(*p*) is identical to the error level in *M*_*γ*_.

As *p* → 1, then *M*_*γ*_ → 1; but very close to this limit, *M*_*γ*_ ≈ 1 is still a good approximation. For the *R*_*k*_ = 10^6^ case, and for *p* = 1.01, KS produces, *M*_*γ*_ ≈ 1.06 (Fig 7 and Table 1). This is an error of 6% which is small considering that it is so close to the singular limit. An error of around 1% away from *p* = 1 is therefore reasonable. In the limit *p* = 3 there is no detectible error in *M*_*γ*_ from KS to three decimal places (Table 2).

KS is an established method used by many researchers in turbulent diffusion studies, as noted in the earlier references in this paper. However, some researchers [23, 33, 38] have argued that KS suffers from systematic erros due to the lack of true dynamical sweep of the small inertial range scales by the much larger energy containing convective scales. However, the author has recently investigated this issue in [32] and shown that such conclusions are unfounded because they were made under the assumption of locality being true. Through an analysis of pairs of trajectories the quantitative errors in KS due to the sweeping effect were proven to be negligible provided that the simulations are carried out in a frame of reference moving with the large scale sweeping flow. The scalings obtained from KS are therefore genuine, and not errors as previously thought.

## 7 Discussion and conclusions

Richardson conceived his scaling law to be applicable to real turbulence, not just a mathematical curiosity. The new theory, developed in [12] and tested numerically here, generalises Richardson’s scaling arguments and is also constructed to be applicable to real turbulence. The fundamentally new concept here is that turbulent pair diffusion is the convolution of local and non-local diffusional processes; and from this idea two limiting cases of non-Richardson pair diffusion regimes has been obtained.

It is important to note that Richardson’s scaling arguments were essentially kinematic in nature, being developed before the age of flow structures and dynamical energy transfer between different scales and intermittency was established. As such, the scaling laws are broadly independent of the precise mechanisms of dynamical energy transfer between different scales, much like the Kolmogorov spectrum itself. This is true also of the new local-non-local theory for the same reasons.

This may also explain why KS works well in the investigation of statistical scaling laws. KS is a Lagrangian model for tubulent diffusion, comparable to non-Markovian stochastic diffusion models. KS cannot account for all aspects of turbulence, like actual energy transfer between different scales, large scale sweeping of smaller scales, and true intermittency. However, as the theory itself is constructed for statistical scaling laws that are not strongly dependent on the precise dynamics, this may not be a critical limitation in the present context. Furthermore, it has been shown in [32] that the sweeping error in KS is negligible if simulations are carried out in the swept frame of reference as has been done here; and at least the energy spectrum of intermittent turbulence can be readily implemented in KS. The results presented here show that in the limit of locality, i.e. small inertial subrange, KS yields the correct Richardson scaling, and in the limit of asymptotically infinite inertial subrange with intermittent-like spectrum we obtain a non-local scaling law which is remarkably close to the revised 1926 data. These results provide some degree of confidence in the fidelity of the KS results; but we will have to wait for DNS or experiments that can interrogate very large inertial subranges for a definitive answer to how realistic KS statistics is of actual turbulence.

The main mathematical hypothesis of this theory is the separation of the inertial subrange in to two wavenumber ranges, which leads to the existence of two broadly independently diffusional processes that produce local and non-local scalings individually, but together they produce scale dependent diffusion coefficients with constant power laws *γ*(*p*)—the actual value of the *γ*(*p*) is dependent upon the inertial range power law ∼ *k*^−*p*^ and also upon the size of the inertial subrange, *R*_*k*_ Eq (1).

The main predictions of the theory are, firstly that in turbulence with generalized energy spectra, *E*(*k*) ∼ *k*^−*p*^, and for asymptotically infinite inertial range, *R*_*k*_ → ∞, the pair diffusion coefficient scales like, $K\left(l,p\right)∼\sigma_{l}^{\gamma (p)}$, with (1 + *p*)/2 < *γ*(*p*) ≤ 2, in the range 1 < *p* ≤ 3, which is intermediate between the purely local and purely non-local scaling power laws. Secondly, for small inertial subranges, there exists quasi-local scaling regimes, with *γ*(*p*) ≈ (1 + *p*)/2, because the non-local range of scales are effectively absent and the entire inertial subrange then acts locally at all separations inside the inertial subrange.

The resuts from the KS simulations reported here confirm all the predictions of the theory. Most importantly, two sets of non-Richardson pair diffusion regimes are observed: (1) non-local regimes for asymptotically infinite inertial subrange, *R*_*k*_ = 10^6^, Figs (1) to (5); and (2) quasi-local regimes for short inertial subrange of *R*_*k*_ = 10^2^, Figs (6) to (10). A smooth transition from small to large subranges is observed in Figs (11) to (14).

The simulation results for *R*_*k*_ = 10^6^ are in good agreement with the revised geophysical data in Fig 1 in [12], $K∼\sigma_{l}^{1.564}$. In the critical range of intermittent spectra, the scalings *γ* are generally within about 1% of 1.564. For *E*(*k*) ∼ *k*^−1.72^, the simulations produced, $K_{\mu_{I}}∼\sigma_{l}^{1.556}$. The equivalent power law scaling in 〈*l*^2^〉 is ∼ *t*^4.505^, Table 1.

In short inertial subranges of size *R*_*k*_ = 10^2^ in the critical range of intermittent spectra, the scalings *γ*(*p*) are generally within about 5% of locality scaling laws. For intermittency observed in real turbulence, *E*(*k*) ∼ *k*^−1.72^, the simulations produce the scaling law, $K∼\sigma_{l}^{1.43}$, and the equivalent power law scaling in 〈*l*^2^〉 is ∼ *t*^3.509^, Table 2.

An important corollory of the current work is that real turbulence with intermittency does *not* contain the classical R-O *t*^3^-regime, even under the locality hypothesis. This is remarkable because the R-O *t*^3^-regime has been much debated for decades and its existance taken for granted.

To date no scaling greater than *t*^3^ inside the inetial subrage has been reported in experiments or in DNS, except for the 1926 dataset—this implies that current laboratory experiments and DNS where pair diffusion has been investigated are still in or below the quasi-locality limit. Indeed, the biggest inertial subranges generated in current DNS is around *R*_*k*_ = 10^2^, which is consistent with our theory and simulations which suggest that this is indeed the lower limit for quasi-local regimes to be observable.

Some results apparently showing pair diffusion scaling greater than ∼ *t*^3^ have been reported in some recent DNS, [39, 40]. However, these results are for small initial separations *l*_0_ ≤ *η* and appear over a short time period after release. The authors themselves note that this is probably due to the influence of the separation in the dissipation range at earlier times—that is, in a DNS which contains a genuine dissipation range, particles that have left the dissipation range continue to be affected by the dissipation range some distance in to the inertial subrange. This is likely a manifestation of the ultra-violet corrections mentioned in the main text above and also in [12]. This should not be confused with genuine inertial range scaling.

Finally, we remark that the concepts investigated here and in [12] could have a significant impact on the general theory of turbulence, evoking some important questions for the future. For example, are long-range correlations in turbulence more significant than previously thought in high Reynolds number turbulence? Could turbulent diffusion be better modelled as a bi-variant process? And, if experiments ultimately do show that the KS results are close to real turbulence statistics, does this mean that the dynamics plays a less important role for lower order Lagrangian statistics?
